# Passive Metasurface Tweezers for Multi‐Scale Orbital Transport and Trapping on Elastic Plates

**DOI:** 10.1002/advs.77631

**Published:** 2026-09-19

**Authors:** Zeyang Bi, Zhiqiang Li, Yunkang Ren, Yugui Peng, Xuefeng Zhu, Liuxian Zhao, Jun Yang, Chuanxing Bi

**Affiliations:** ^1^ Institute of Sound and Vibration Research Hefei University of Technology Hefei China; ^2^ State Key Laboratory of Acoustics and Marine Information Institute of Acoustics Chinese Academy of Sciences Beijing China; ^3^ College of Underwater Acoustic Engineering Harbin Engineering University Harbin China; ^4^ School of Physics and Innovation Institute Huazhong University of Science and Technology Wuhan China; ^5^ School of Electronic Electrical and Communication Engineering University of Chinese Academy of Sciences Beijing China

**Keywords:** cross‐scale manipulation, flexural waves, flexural‐wave vortex, metasurface, orbital angular momentum, particle manipulation

## Abstract

Acoustic manipulation on solid surfacesis often constrained by limited wave‐field reconfigurability and reliance on complex active excitation, hindering the simultaneous realization of particle confinement and controlled rotation. Here, we present a passive, subwavelength‐encoded platform that enables cross‐scale confinement and orbital manipulation on an open thin plate under single‐channel power‐source excitation. By integrating graded‐phase, high‐transmission labyrinthine superstructures, the platform enables precise spatial phase modulation without electronic modulation. The system preserves flexural wave vortex fields with well‐defined phase singularities over a broad operating bandwidth (∼8 kHz). To elucidate the underlying physics, a phenomenological force framework is established: orbital angular momentum induced by azimuthal phase gradients drives circumferential motion, while amplitude‐gradient‐induced asymmetric interactions provide an effective radial confinement force. This synergistic mechanism facilitates multi‐scale manipulation of objects spanning from sub‐millimeter particles to lightweight centimeter‐scale structures. Furthermore, the vortex chirality can be reversed solely via structural inversion, revealing a passive control strategy governed by spatial symmetry. This work establishes a versatile framework for integrated surface‐wave manipulation, with potential applications in lab‐on‐a‐surface systems.

## Graphical abstract

Passive labyrinthine phase encoding converts single‐channel excitation into localized flexural‐wave vortices with programmable orbital angular momentum. The resulting vortex fields enable cross‐scale particle manipulation, including orbital transport, central confinement, and rotational actuation. Frequency tuning adjusts the vortex‐core size, while reversing the topological charge switches the orbital direction, providing a compact and tunable manipulation platform.

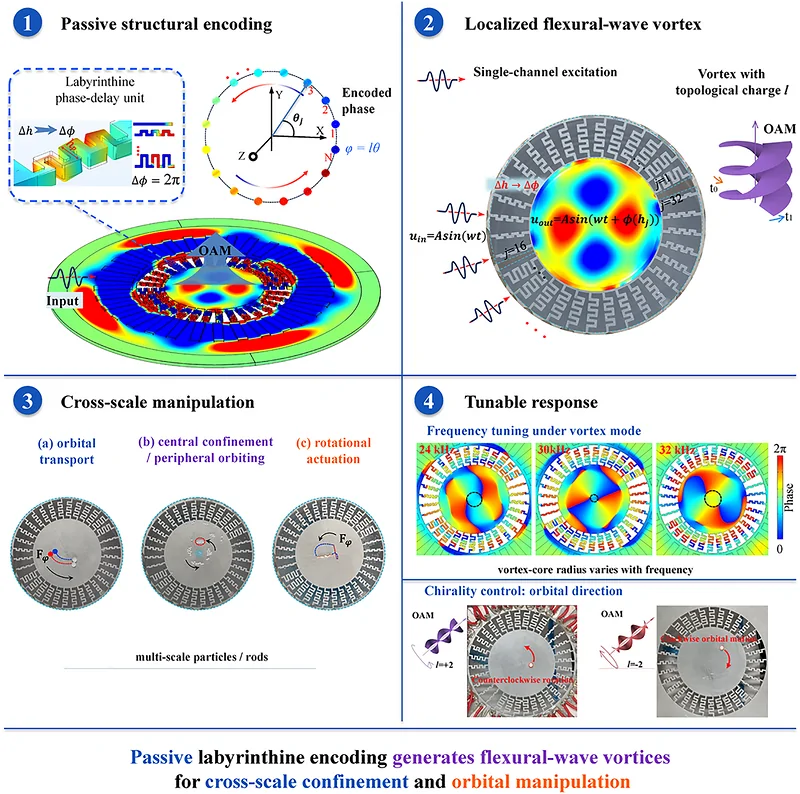

## 1 Introduction

Precise, contactless manipulation of matter across multiple length scales is a pivotal capability in modern science and engineering, driving transformative advances in biophysics, tissue engineering, and microrobotics [1, 2, 3]. To achieve such manipulation, a variety of physical fields have been developed as “virtual tweezers” for the capture, transport, and actuation of micro‐objects. Notably, optical tweezers utilize optical momentum and scattering forces for high‐resolution trapping and manipulation [4, 5], magnetic systems enable remote actuation and torque transfer through externally applied magnetic fields [6, 7], and electric‐field‐based techniques exploit applied electric fields to achieve localization and directed transport of micro‐objects through electrostatic and electrokinetic effects [8, 9]. Among various modalities, acoustic manipulation stands out as an exceptionally attractive strategy due to its noninvasive nature, superior biocompatibility, and label‐free operation, particularly its ability to penetrate optically opaque media with significant depth [10, 11, 12, 13, 14, 15, 16]. Specifically, surface acoustic wave (SAW)‐based technologies have established a versatile foundation for lab‐on‐a‐chip applications, enabling high‐efficiency patterning of microparticles and cells, as well as localized acoustofluidic regulation [17, 18, 19, 20, 21]. Nevertheless, many existing acoustic manipulation schemes still rely on relatively static trapping configurations, such as standing‐wave pressure nodes or single‐beam acoustic traps, which are effective for basic trapping and patterning but limited in continuous transport, rotational actuation, and flexible wavefront shaping [22, 23, 24]. More advanced manipulation often requires actively synthesized traveling or vortex‐like acoustic fields, frequency switching, or multimodal excitation, thereby increasing system complexity and control demands [25, 26, 27, 28]. Achieving compact surface‐based manipulation with simplified excitation and controlled orbital dynamics therefore remains a significant challenge.

A major limitation of these approaches is the difficulty of achieving continuous rotational manipulation together with independent control of orbital dynamics on compact solid platforms. Structured wavefields carrying orbital angular momentum (OAM) offer a route beyond this limitation. Originally established in optical systems [29, 30, 31, 32], OAM has been extended to acoustics, where wavefields with azimuthal phase dependence $e^{\mathrm{il}\theta }$ exhibit phase singularities and helical wavefronts [33, 34, 35]. These features enable the transfer of angular momentum to matter, giving rise to mechanical torque, orbital transport, and controlled rotational motion [36, 37, 38, 39, 40]. Recent advances have correspondingly extended acoustic manipulation beyond trapping toward transport, cargo delivery, and three‐dimensional dynamic trajectories [12, 41, 42]. Recent studies have further demonstrated vortex‐based manipulation and size‐dependent trapping in acoustic vortex fields [34, 36, 43, 44]. In parallel, active holographic synthesis, phased‐array trapping, and time‐multiplexed acoustic tweezers have enabled increasingly sophisticated contactless manipulation in fluids [45, 46]. Yet stable and integrated manipulation on solid elastic platforms, especially thin plates, remains difficult. More generally, existing vortex‐based acoustic manipulation schemes often depend on multichannel phased arrays, traveling/standing‐wave switching, holographic synthesis, or dynamically reconfigurable wave‐field engineering. This increases system complexity and sensitivity to phase mismatch and modal instability. In flexural‐wave systems, strong dispersion and multimodal coupling further hinder the preservation of vortex phase characteristics across frequency. On liquid‐loaded or vibration‐driven solid surfaces, streaming‐dominated migration, anti‐nodal accumulation, and mode‐sensitive collective motion can further compromise deterministic orbital control [21, 24, 36, 47].

Here, we address these challenges by introducing a subwavelength‐encoded passive superstructure that generates flexural‐wave vortex fields with structurally encoded phase winding on an open thin plate under single‐channel excitation. Unlike traveling/standing‐wave switching, time‐multiplexed trapping, or multi‐frequency dynamically reconfigurable wave synthesis [45, 46, 48, 49, 50], our approach uses real‐space structural phase encoding to reconstruct the target vortex field through deterministic phase accumulation. This strategy avoids multichannel electronic synchronization and instead establishes a compact passive route to vortex‐wave generation on solids. The resulting wavefield exhibits a spatially decoupled manipulation mechanism: azimuthal phase gradients drive orbital motion, whereas amplitude gradients provide radial confinement. In this way, the platform enables surface‐based confinement and orbital manipulation across multiple length scales, while the frequency‐dependent measurements distinguish phase‐singularity preservation from variations in manipulation performance.

## 2 Results

### 2.1 Design Principle and Structural Encoding of Flexural Wave Vortices

**FIGURE 1:**
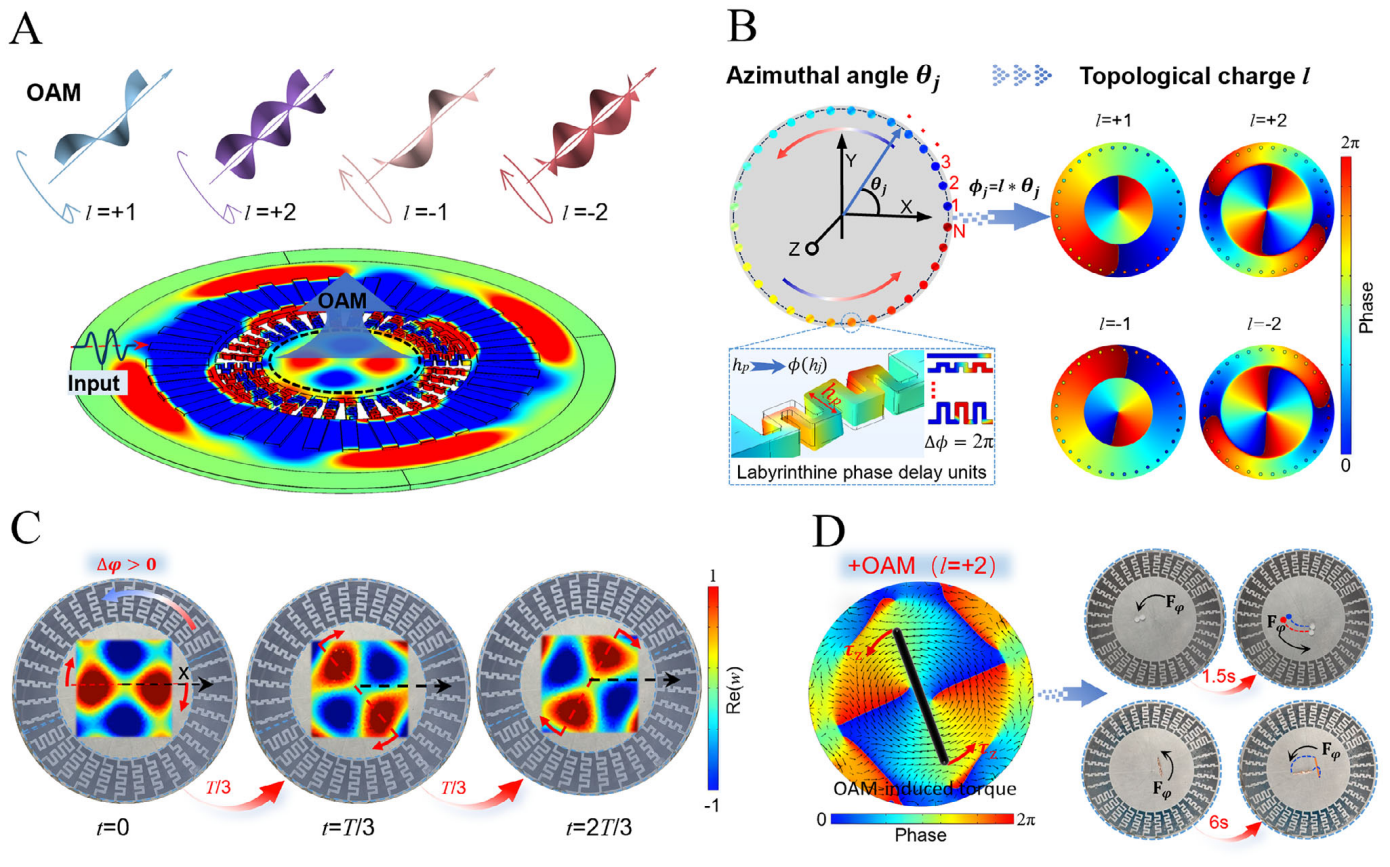
Structurally encoded flexural topological vortex generation and cross‐scale object manipulation. (A) Platform principle: A single‐channel harmonic source excites an elastic thin plate patterned with subwavelength labyrinthine phase‐shifting units. The geometric phase modulation passively generates flexural wave vortices carrying the OAM. (B) Structured phase encoding: The azimuthal phase profile ϕ(θ)=lθ is encoded into the geometric gradients of the labyrinthine units, enabling the generation of vortex fields with a predefined topological charge *l* under single‐source excitation. (C) Time‐resolved displacement evolution: Snapshots of the real displacement 𝑅𝑒(𝑤) at 𝑇/3 intervals. The quasi‐stationary amplitude envelope combined with rotating displacement patterns indicates continuous azimuthal phase evolution and reveals the intrinsic rotational nature of the vortex field. (D) Physical mechanism and manipulation: Momentum transfer from the vortex field exerts an acoustic torque $T_{z}$ on objects. Under surface constraints, OAM is converted into a tangential driving force $F_{\varphi }$, enabling controlled orbital rotation of both spherical particles and elongated structures across multiple length scales.

To control flexural vortex wave fields and surface particle motion, we developed a passive structurally encoded platform based on a functionalized elastic thin plate. As illustrated in Figure 1A, a single‐channel harmonic source excites the plate through an annular array of PZT elements, while subwavelength labyrinthine units are patterned in the wave‐propagation region. A detailed schematic of the device architecture, including the PZT array and its electrical interconnection, is provided in Figure S1. The odd‐numbered and even‐numbered PZT elements are arranged as two interleaved parallel groups. Both groups are connected to the same output terminals of a single‐channel power amplifier and receive the same harmonic voltage signal, providing spatially distributed, nominally in‐phase excitation without independently programmed electronic phase delays. The labyrinthine units act as local phase shifters, imposing spatially varying phase delays on the propagating flexural waves through geometry‐induced effective refractive‐index modulation. Therefore, unlike active phased arrays that rely on multi‐channel electronic phase control, the proposed platform generates the required azimuthal phase accumulation through passive structural encoding, enabling the generation of localized flexural OAM fields within a compact footprint.

The topological characteristics of the vortex field are determined by its azimuthal phase distribution. As shown in Figure 1B, the encoded phase profile follows ϕ(θ)=lθ, where 𝜃 is the azimuthal angle and 𝑙 is the topological charge. The sign of 𝑙 is determined by the direction of phase winding relative to the prescribed angular coordinate: ϕ increasing with θ yields l>0, whereas ϕ decreasing with θ yields l<0. By translating a discrete phased‐array model onto continuous labyrinthine geometries, we realized a discrete‐to‐continuous structural phase encoding strategy. This allows the metastructure to generate vortex fields with predefined topological charges solely through structural encoding under single‐channel power‐source excitation, with chirality intrinsically locked into the spatial phase arrangement.

Time‐resolved analysis of the displacement field reveals the underlying physics of the flexural wave vortex. The out‐of‐plane vibration displacement field was experimentally measured using a scanning laser Doppler vibrometer (SLDV). Snapshots of the instantaneous out‐of‐plane displacement at *t*_0_, *t*_0_ + *T*/3, and *t*_0_ + 2*T*/3 (Figure 1C) exhibit a rotating wavefront. While the amplitude envelope remains stationary—with a central low‐amplitude region and the phase field contains a singularity at the vortex core, the displacement pattern evolves continuously in the azimuthal direction with time (Movie S1), indicating a persistent vortex‐like propagation behavior associated with the OAM.

This encoded OAM serves as the primary driver for particle manipulation. As depicted in Figure 1D, for l>0, the positive azimuthal phase gradient induces a tangential momentum flow in the direction set by the phase winding, exerting an acoustic torque that drives orbital motion. Simultaneously, the radial amplitude gradient—defined by the central low‐amplitude region and the surrounding high‐intensity annulus—creates an effective potential well for radial confinement. For elongated objects, this dual‐force mechanism enables preferential localization of one end near the vortex core while the other undergoes orbital rotation. Notably, a mirror inversion of the metastructure reverses the phase gradient, thereby switching the OAM sign and rotation direction without altering the excitation frequency. This symmetry‐based strategy eliminates the need for active wavefront engineering and establishes a passive route for surface acoustic manipulation across multiple length scales.

### 2.2 Mechanism of Particle Manipulation

**FIGURE 2:**
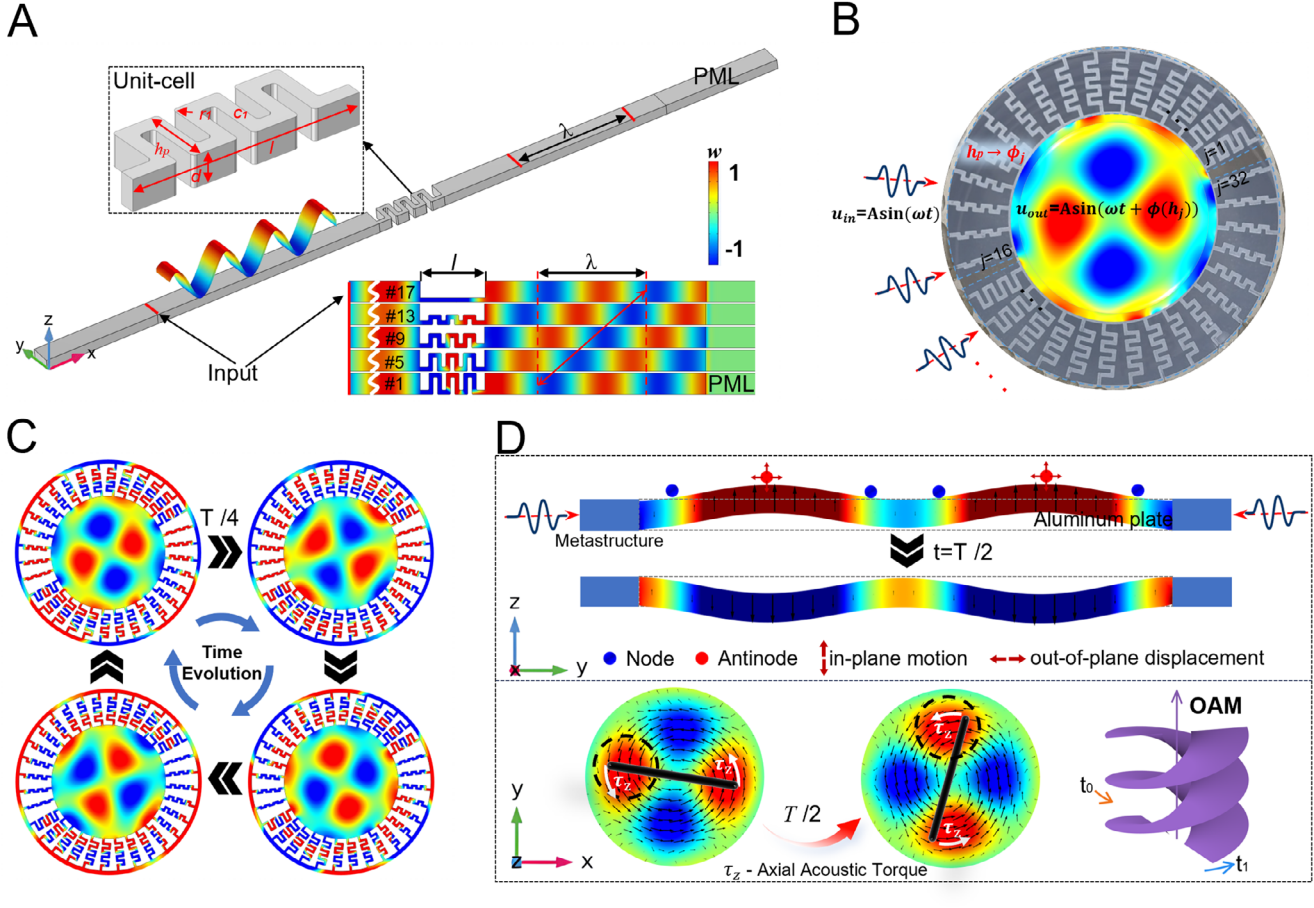
Generation and dynamical characteristics of structurally encoded flexural wave vortices. (A) The structural height $h_{p}$ modulates the effective phase response of the unit, introducing a geometry‐driven phase delay $\phi (h_{p})$. This establishes a deterministic mapping between geometry and phase, $h_{p}\to \phi \left(h_{p}\right)$. (B) Topological phase encoding and vortex formation. Under harmonic excitation $u_{\mathrm{in}}=A\sin\left(\omega t\right)$, the labyrinthine metastructure spatially modulates the propagation phase, producing a continuous 2𝜋 azimuthal phase accumulation at the output and generating a flexural wave vortex field with a prescribed topological charge 𝑙. (C) Time‐resolved evolution of the vortex field. Snapshots of the displacement field over one oscillation period show continuously rotating spatial patterns, while the amplitude envelope remains nearly stationary. This behavior indicates a phase‐driven rotating wavefront rather than modal switching. (D) Mechanisms of particle manipulation. The amplitude gradient induces a radial drift force $F_{r}$ via vibration‐induced rectification, enabling radial confinement. Meanwhile, the orbital angular momentum $L_{z}$ generates a torque $\tau_{z}$ through momentum transfer, which drives sustained orbital motion.

The generation of flexural wave vortices originates from the passive phase modulation programmed into the labyrinthine units. As illustrated in Figure 2A, each unit tailors its effective phase response through its specific geometry, establishing a deterministic mapping $h_{p}\to \phi \left(h_{p}\right)$. For a unit at azimuthal position $\theta_{j}$, the structural height $h_{j}$ is designed to satisfy the phase‐matching condition:

(1) $$\phi \;\left(h_{j}\right)=\frac{2\pi lj}{N}$$

where l is the topological charge, and N=32 is the number of unit cells. Full‐wave simulations at 30 kHz confirm that varying $h_{p}$ from 1 mm to 4.95 mm achieves full 2π phase coverage with high transmission efficiency (Figure S2D). This structural encoding imposes a continuous azimuthal phase accumulation (Figure 2B), resulting in a vortex field expressed as:

(2) $$W\;\left(r,\varphi \right)=A_{l}\;J_{l}\left(k_{b}r\right)e^{il\varphi }$$

with the corresponding time‐domain displacement given by $w\left(r,\varphi ,t\right)=R_{e}\left\{W\left(r,\varphi \right)e^{i\omega t}\right\}$. Here, (r,φ) denote the radial distance and azimuthal angle, respectively, $J_{l}\left(\cdot \right)$ is the l‐th order Bessel function of the first kind, and $A_{l}$ is the complex mode amplitude. Time‐resolved analysis (Figure 2C) shows that the instantaneous displacement w(r,φ,t) rotates continuously within one period, whereas the amplitude envelope |W| remains stationary. This confirms that the observed vortex originates from a phase‐driven rotating wavefront rather than from modal switching.

To elucidate the mechanical origin of the observed manipulation, we established a phenomenological force framework in which particle dynamics arise from the combined action of radial confinement and azimuthal transport. Drawing upon the mechanics of structured waves [31, 35], the total force F exerted on a particle can be partitioned into an effective gradient force and an OAM‐induced driving force, revealing the decoupled nature of trapping and orbital motion.

As illustrated in Figure 2D, the flexural vibration of the plate exhibits a structured amplitude landscape with stationary low‐vibration regions and periodically oscillating high‐amplitude regions. This high‐frequency motion induces asymmetric contact dynamics between the surface and the particle. As theoretically explored in vibrational acceleration models [51], the localized acceleration of the plate creates an effective dynamic slope that drives particles toward specific equilibrium positions. From a statistical mechanics perspective, these equilibrium sites correspond to the stochastic stability of nodal sets [52], where random fluctuations in particle trajectories are minimized. Phenomenologically, this interaction creates an effective radial trapping force $F_{r}$, whose radial component can be written as:

(3) $$F_{r}=-\alpha \frac{\partial {\left|W\right|}^{2}}{\partial r}$$

where α is an effective phenomenological coupling coefficient introduced to describe the combined effects of elastic recovery, frictional dissipation, and particle–surface interactions during repeated collisions. The value of α is not independently calibrated in the present experiments and is introduced as an effective parameter to characterize the radial confinement tendency. Near the vortex core, where the vibration amplitude rises monotonically from zero, this gradient force naturally favors radial confinement toward the low‐excitation central region.

Simultaneously, the phase gradient ∇ϕ inherent in the flexural vortex creates a persistent circulating energy flux, corresponding to a non‐zero canonical momentum density. Following structured wave principles [31, 35], the time‐averaged in‐plane momentum density $P_{\varphi }$ is purely azimuthal:

(4) $$P_{\varphi }=\frac{1}{2}\;\rho h\omega \frac{l}{r}{\left|A\left(r\right)\right|}^{2}{\hat{e}}_{\varphi }$$

where ρ and h denote the density and thickness of the plate, respectively, and $A\left(r\right)=A_{l}J_{l}\left(k_{b}r\right)$. The direction of this momentum flux is intrinsically locked to the azimuthal unit vector ${\hat{e}}_{\varphi }$, originating from the helical phase gradient $\nabla \left(l\varphi \right)=\left(l/r\right){\hat{e}}_{\varphi }$. This relation indicates that the circulating momentum flow is fundamentally governed by the sign of the topological charge l. The corresponding orbital angular momentum (OAM) density L perpendicular to the plate is:

(5) $$L_{z}=r\times \;P_{\varphi }=\frac{1}{2}\;\rho h\omega l{\left|A\left(r\right)\right|}^{2}{\hat{e}}_{\varphi }$$

showing that the direction of orbital angular momentum transfer is set by the sign of the topological charge l [32, 53]. Through a momentum‐transfer mechanism—analogous to the mechanical torque observed in acoustic vortices [37, 54]—this stored OAM is converted into a mechanical driving action. Under surface‐contact conditions, this driving action can be phenomenologically described by an effective tangential force density $F_{\varphi }$:

(6) $$F_{\varphi }=\eta \;P_{\varphi }=\frac{1}{2}\;\eta \rho h\omega \frac{l}{r}{\left|A\left(r\right)\right|}^{2}$$

where η represents the effective phenomenological momentum‐transfer efficiency. This expression shows that the azimuthal driving force density is jointly determined by the local wave intensity and the helical phase gradient. Although the 1/r factor suggests an enhanced phase gradient near the center, the field amplitude simultaneously vanishes at the singularity for |l|≥1 $\left({\left|A(r)\right|}^{2}\propto r^{2|l|}\right)$. As a result, the tangential driving force smoothly tends to zero as r→0, while reaching an optimal balance for orbital transport in the finite annular region surrounding the vortex core.

For an extended object occupying an area S, the resulting mechanical torque about the vortex axis is the integral of this force density:

(7) $$\tau_{z}=\int \;\;\;\int rF_{\varphi }dS=\frac{1}{2}\eta \rho h\omega l\int \;\;\;\int {\left|A\left(r\right)\right|}^{2}dS)\;$$

Equation 7 reveals the essential mechanism of passive orbital manipulation: the magnitude of the torque is governed by the spatially intercepted wave intensity, whereas its direction is determined solely by the sign of l. Unlike traditional Chladni particles that remain stationary at nodal regions [52], the symmetry‐breaking helical phase of the vortex field enables persistent orbital transport, while the angular momentum transfer mechanism remains applicable across multiple scales. In our system, this vortex torque enables a passive manipulation paradigm for objects spanning from sub‐millimeter particles to centimeter‐scale structures, with the rotation direction determined by the sign of the topological charge l.

### 2.3 Frequency Detuning Effects on Vortex‐Core Evolution and Particle Manipulation Performance

**FIGURE 3:**
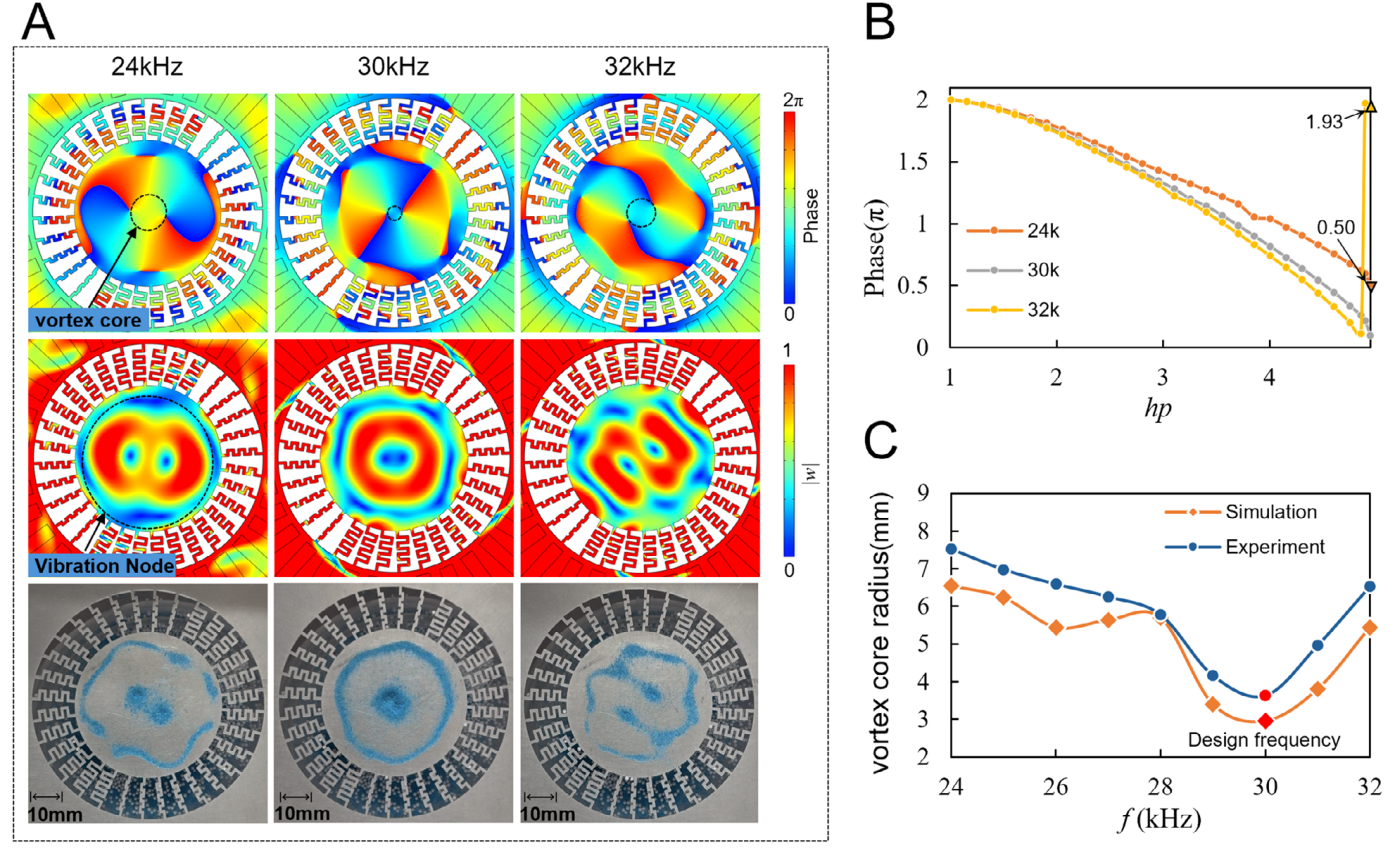
Spectral characterization of the encoded flexural‐wave vortex under frequency detuning. (A) Numerical phase distributions and normalized out‐of‐plane displacement amplitude fields |w| of the encoded vortex field at representative frequencies of 24, 30, and 32 kHz. The phase distributions show that the azimuthal phase winding and the central phase singularity remain observable under frequency detuning. The bottom row shows experimentally observed fine‐particle accumulation patterns (Movie S2). Because the fine sand‐like particles preferentially settle in the low‐displacement region, these patterns are used to visualize and estimate the effective experimental vortex‐core region rather than to quantify dynamic orbital manipulation. (B) Frequency‐dependent phase‐delay response of the labyrinthine meta‐units as a function of the structural height $h_{p}$. At the design frequency of 30 kHz, the encoded units provide the desired 2π phase‐closure condition. In contrast, operation at 24 and 32 kHz leads to phase under‐compensation and over‐compensation, respectively, due to structural dispersion. (C) Frequency dependence of the vortex‐core radius $r_{c}$. The numerical core radius was extracted from the simulated phase singularity and amplitude‐null distribution, while the experimental effective core radius was estimated from the fine‐particle accumulation patterns in (A). The minimum vortex‐core radius occurs near the design frequency of 30 kHz. Off‐design operation leads to core expansion because of dispersion‐induced phase mismatch. These results demonstrate broadband persistence of the vortex‐field phase singularity, but they also reveal that the vortex‐core size is frequency dependent.

To evaluate the frequency response of the structurally encoded flexural wave vortices under experimental conditions, an integrated synchronous excitation platform consisting of 32 piezoelectric patches was constructed (Figure S3), driven by a single signal source without external phase modulation. The design operating frequency of the metasurface is 30 kHz (corresponding to a flexural wavelength of λ  =  2.5 cm), at which the labyrinthine structural units provide precise optimized azimuthal phase compensation to satisfy the target 2π phase closure condition. This induces strong destructive interference near the geometric center, yielding the minimum vortex core radius $r_{c}$ across the tested spectrum (Figure 3C), which corresponds to the strongest experimentally observed radial confinement tendency and a highly concentrated particle orbital response. As illustrated in Figure 3A, when the operating frequency is detuned within the range of 24–32 kHz, both simulated and experimentally measured displacement fields maintain discernible azimuthal phase winding and a central low‐displacement region, demonstrating that the fundamental phase singularity persists within this bandwidth. It is worth emphasizing that the ‘broadband robustness’ discussed herein specifically refers to the preservation of the wave field's topological features—the persistence of the phase singularity and the vortex wavefront under detuning—rather than implying that the vortex‐core profile, orbital radius, particle velocity, or manipulation stability remain rigidly invariant across the entire spectrum. Therefore, the preservation of the wave‐field phase singularity should be distinguished from the frequency‐dependent manipulation performance.

Despite this phase‐singularity robustness [55], the morphology of the vortex core exhibits a pronounced frequency dependence governed by the dispersion‐driven phase response of the labyrinthine structural units [56]. As shown in Figure 3B, the phase delay of the structural units is governed by their equivalent propagation paths and the flexural wavelength λ(f). When the operating frequency deviates from the 30 kHz design center, a phase mismatch occurs between the actual phase compensation and the target vortex phase distribution. At the design frequency of 30 kHz, the structural units provide nearly complete 2π phase coverage over the height range of $h_{p}$ = 1−4.95 mm, indicating near‐target phase compensation. When the frequency is shifted downward to 24 kHz, the increased flexural wavelength causes insufficient phase delay, resulting in phase under‐compensation. In this case, the phase coverage is reduced to approximately 0−1.5π, corresponding to a phase‐coverage deficit of 0.5π relative to the ideal 2π target, i.e., about 90°. Conversely, when the frequency is shifted upward to 32 kHz, the shortened wavelength enhances the phase delay, leading to phase over‐compensation. The phase coverage increases to approximately 0−2.07π, corresponding to an excess phase coverage of 0.07π, i.e., about 12.6°.

This dispersion‐induced phase‐coverage deviation weakens the ideal destructive interference at the vortex center and introduces residual coherent components. As a result, the vortex core radius exhibits a “U‐shaped” variation with frequency, reaching a minimum near the 30 kHz design frequency and expanding under both low‐ and high‐frequency detuning conditions, as shown in Figure 3C. In the experiments, the fine sand particles shown in the bottom row of Figure 3A served as visual tracers for the low‐displacement region (Movie S2). After pixel calibration using the 10 mm scale bar, the central particle‐accumulation region was extracted and fitted with an ellipse. The effective experimental vortex core radius was defined as half of the major axis of the fitted ellipse. As depicted in Figure 3C, the measured effective vortex core radius agrees well with the simulated trend, confirming the systematic evolution of the vortex core profile under frequency detuning.

To quantitatively analyze the effects of frequency detuning on particle manipulation performance, orbital manipulation experiments were performed at 24, 30, and 32 kHz under a constant peak voltage of 190 V (Movie S3). Population statistical analysis—utilizing frame‐by‐frame centroid detection and short‐trajectory velocity estimation within a selected 30–40 s analysis window (summarized in Figure S5 and Table S1)—reveals that while the preserved phase singularity provides the wavefield baseline for orbital motions, the manipulation performance exhibits a prominent frequency dependence. Here, the counterclockwise direction is defined as positive and the clockwise direction as negative. At the 30 kHz design frequency, the system delivers the optimal overall manipulation performance, featuring highly concentrated circular orbits with an average orbital radius $R_{\mathrm{mean}}$ ≈ 10.64 mm, a trajectory standard deviation $R_{\mathrm{std}}$ ≈ 5.25 mm, and peak average tangential velocity $v_{\theta ,\mathrm{mean}}$ ≈ 8.50 mm/s and angular velocity $w_{\mathrm{mean}}$ ≈ 1.09 rad/s. When detuned downward to 24 kHz, despite a smaller $R_{\mathrm{std}}$, the average orbital radius expands significantly (to ∼13.95 mm) while the angular velocity drops by 46.4% compared to the design baseline, indicating that the particles undergo slow bounded rotations on a wider orbit. When detuned upward to 32 kHz, the manipulation performance further degrades; compared to the 30 kHz baseline, $R_{\mathrm{mean}}$ increases by 22.2% (to ∼ 13.00 mm), the trajectory deviation $R_{\mathrm{std}}$ surges by 39.6%, and the average tangential and angular velocities drop drastically by 50.1% and 46.3%, respectively (detailed frame‐specific and representative state statistics are compiled in Table S1). Adopting the maintenance of a bounded orbital motion within this window as the success criterion, the bounded trapping capability drops notably upon detuning.

### 2.4 Dynamical Characterization of Multi‐Scale Manipulation

To evaluate the manipulation capability of the structurally encoded vortex field across different object sizes and geometries, experiments were performed using millimeter‐scale particles, submillimeter particles, and an elongated lightweight object. The tunability of the present passive platform arises from the fixed structural encoding combined with external adjustment of the excitation frequency and voltage, rather than from real‐time programmable control. The reconstructed vortex field provides two spatially distinct mechanical effects: radial confinement associated with the amplitude gradient and azimuthal transport associated with the phase gradient and circulating energy flux.

**FIGURE 4:**
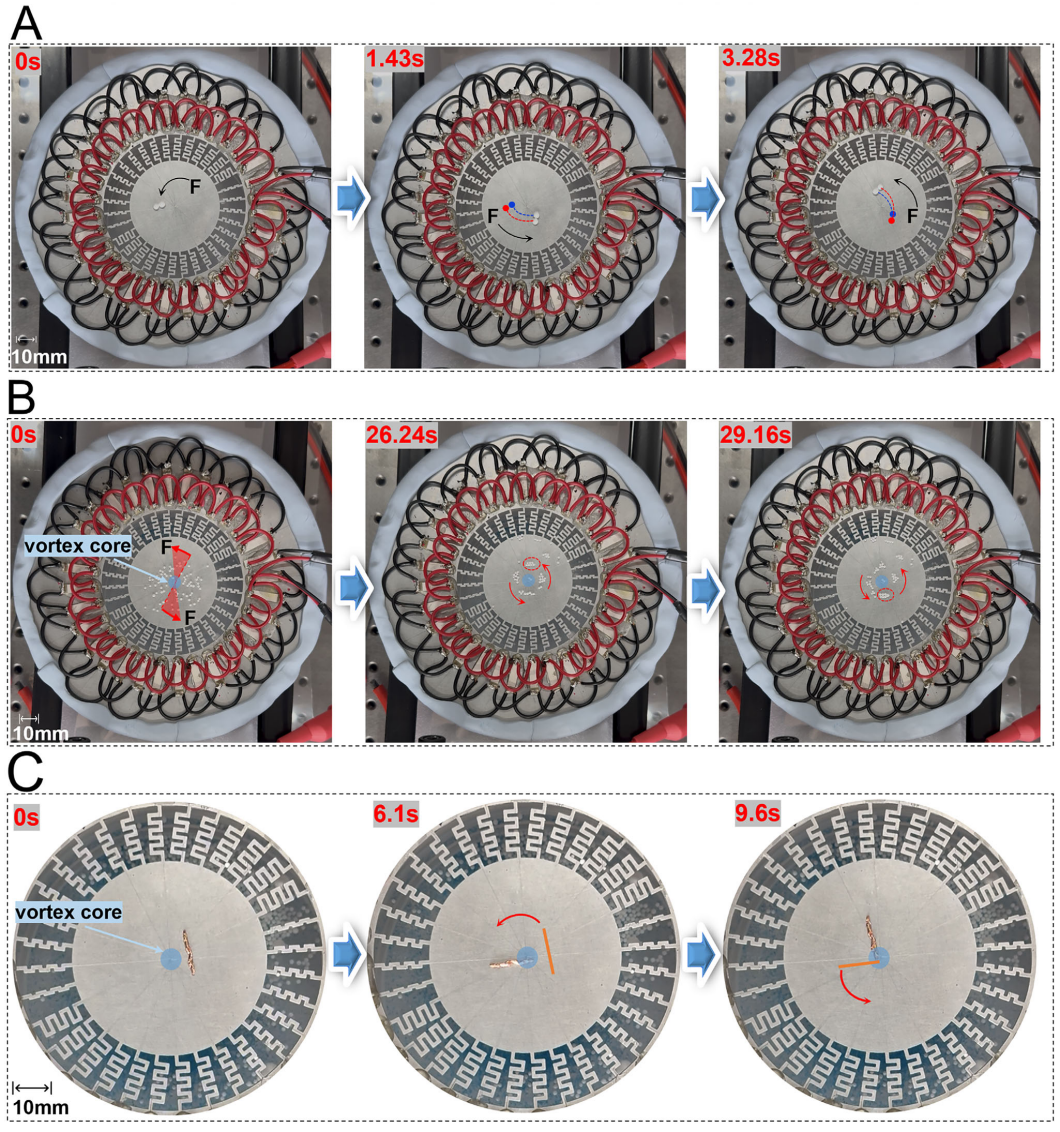
Experimental demonstration of multiscale object manipulation under 30 kHz flexural‐wave vortex excitation. (A) Time‐lapse snapshots of millimeter‐scale foam particles with diameters of 3–5 mm undergoing sustained orbital motion around the vortex center. The bounded circular trajectories are consistent with azimuthal momentum transfer from the flexural‐wave vortex to the particles, enabling continuous orbital transport. (B) Manipulation of submillimeter particles with diameters of 0.3–0.6 mm. Particles near the vortex core migrate toward and remain localized within the central low‐amplitude region, whereas particles in the surrounding region undergo persistent orbital transport. The two motion regimes reflect the combined effects of radial amplitude‐gradient confinement and azimuthal phase‐gradient propulsion. (C) Dynamic manipulation of a lightweight rolled copper‐foil rod with a typical length of approximately 1.5 cm. One end remains near the low‐amplitude vortex‐core region, while the rod body overlaps with the surrounding azimuthal momentum‐transfer region, producing a net torque and pivot‐like rigid‐body rotation. This result demonstrates the manipulation of an elongated, non‐spherical object.

Figure 4A shows the orbital motion of millimeter‐scale foam particles with diameters of 3–5 mm (0.12λ −0.20λ) under continuous 30 kHz excitation. The particles remain within bounded orbital regions and undergo sustained circumferential motion around the vortex center. This behavior is consistent with the transfer of azimuthal momentum from the flexural‐wave vortex to the particles, whereby the circulating wave‐energy flux provides sufficient tangential driving to overcome interfacial resistance and maintain orbital transport (Movie S4).

Submillimeter particles with diameters of 0.3–0.6 mm (∼0.02λ) exhibit two distinct motion regimes (Figure 4B). Particles initially located near the vortex core migrate toward and remain confined within the central low‐amplitude region, whereas particles in the surrounding region follow persistent orbital trajectories. Within the phenomenological mechanical description adopted here, these behaviors arise from the combined action of radial amplitude‐gradient confinement and azimuthal phase‐gradient propulsion. The coexistence of central confinement and peripheral orbital transport demonstrates spatially differentiated particle manipulation within a single vortex field.

The manipulation capability was further examined using a lightweight rolled copper‐foil rod with a typical length of approximately 1.5 cm (∼0.6λ) (Figure 4C). During excitation, one end of the rod remains near the low‐amplitude vortex‐core region, while the remaining portion overlaps with the surrounding high‐amplitude annular region and receives stronger azimuthal momentum transfer. This spatially nonuniform interaction generates a net torque, resulting in pivot‐like rigid‐body rotation around the vortex center. Because the rolled copper‐foil rod has a non‐ideal surface morphology, variations in interfacial friction and contact conditions may affect the rotational rate. However, these factors mainly influence the quantitative rotation dynamics, while the rotation chirality remains determined by the sign of the vortex topological charge. This response demonstrates that the vortex field can manipulate not only approximately spherical particles but also elongated objects with strongly anisotropic geometries.

Overall, the observed confinement and rotational dynamics are qualitatively consistent with the proposed mechanical picture. To provide quantitative support, particle‐center trajectories were extracted from 40‐s experimental recordings, from which the orbital radius, angular velocity, tangential velocity, and normalized radial fluctuation were evaluated. Voltage‐dependent responses, trapping probabilities, and repeated‐trial results for particles of different sizes are provided in Supporting Information Section VII, with representative voltage‐dependent orbital motions shown in Movie S5. These measurements show that increasing the excitation voltage generally enhances azimuthal transport, while the probability of sustained orbital confinement decreases, revealing a trade‐off between transport speed and confinement reliability.

Furthermore, leveraging the topological‐charge encoding mechanism, we realize passive chirality control for particle manipulation. Reversing the encoded topological charge (from l=+2 to l=−2) reverses the azimuthal phase gradient [57], leading to an opposite direction of OAM‐associated momentum transfer. Experimental observations and Movie S6 confirm that this structural chirality inversion reverses the rotation direction of manipulated particles while maintaining the observable vortex‐wave characteristics.

**FIGURE 5:**
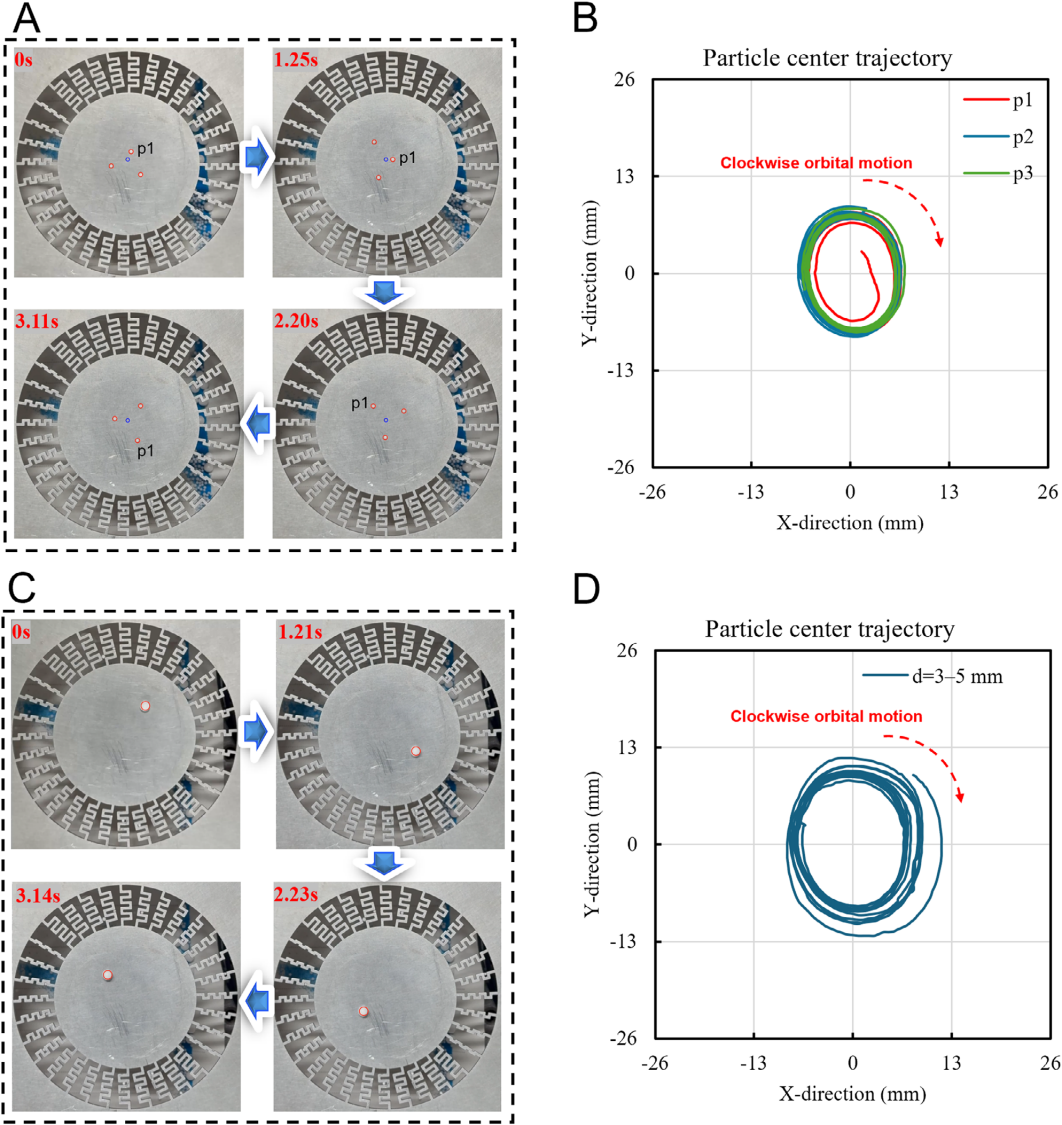
Quantitative verification of clockwise orbital motion under negative‐topological‐charge vortex excitation. (A) Time‐lapse experimental snapshots of three small particles with diameters of 0.3–0.6 mm under negative‐topological‐charge vortex excitation (l=−2), extracted from Movie S6, showing synchronized clockwise orbital motion around the vortex center. (B) Extracted particle‐center trajectories over a 40 s recording period for the three small particles (p1, p2, and p3), revealing bounded closed‐loop orbits with a consistent clockwise rotation direction. (C) Time‐lapse experimental snapshots of a large particle with a diameter of 3–5 mm under the same l=−2 vortex excitation (Movie S6), demonstrating clockwise orbital motion around the vortex center. (D) Extracted center trajectory of the large particle over 40 s, confirming sustained bounded clockwise orbital transport. These results demonstrate that reversing the sign of the encoded topological charge reverses the rotation direction while maintaining vortex‐induced orbital manipulation across different particle sizes.

To quantitatively verify this chirality‐dependent manipulation, we further performed particle trajectory analysis under a negative topological charge (l=−2). As shown in Figure 5A, three small particles with diameters of 0.3–0.6 mm exhibit synchronized clockwise orbital motion around the vortex center. The corresponding particle‐center trajectories extracted from video tracking (Figure 5B) reveal bounded closed‐loop orbits, confirming sustained vortex‐induced orbital transport. Furthermore, a larger particle with a diameter of 3–5 mm also undergoes clockwise orbital motion under the same negative‐chirality vortex excitation (Figure 5C). The extracted trajectory in Figure 5D demonstrates that the OAM‐mediated rotational mechanism remains effective for objects with different size scales. The corresponding angular velocity and tangential velocity responses under different excitation voltages are further quantified in Figure S8, confirming the voltage‐dependent rotational response under reversed vortex chirality.

These results demonstrate that the orbital rotation direction is governed by the sign of the encoded topological charge, whereas the radial confinement behavior remains determined by the amplitude‐gradient‐induced interaction. Therefore, passive structural chirality encoding provides an effective strategy for controlling particle rotation direction without requiring active electronic phase modulation, thereby expanding the functionality of acoustic manipulation systems through passive wave‐field encoding [58].

## 3 Discussion

This study elucidates a paradigm for flexural wave vortex synthesis through passive structural encoding, marking a transition from active phased modulation [45, 46] toward a structure‐mediated wave‐field engineering strategy. By embedding complex azimuthal phase gradients directly into the geometric topography of the metasurface, we achieve targeted wave‐field synthesis under simplified excitation. This approach effectively decouples wave manipulation from electronic complexity, circumventing the circuit‐level intricacies and phase‐synchronization errors inherent in active phased arrays, thereby improving system compactness and simplifying operational control.

Beyond mere simplification, the system's dynamics are governed by the azimuthal phase‐closure condition rather than narrow‐band resonances. Consequently, while structural dispersion triggers phase compensation shifts—manifesting as the characteristic U‐shaped evolution of the vortex core radius—the phase singularity remains preserved over the investigated frequency range. This behavior reflects the structural encoding capability of the platform and highlights the distinction between topological persistence of the wave field and frequency‐dependent variations in manipulation performance [59], rather than providing a direct solution to the frequency sensitivity of acoustic tweezers.

Furthermore, by harnessing vortex waves, we demonstrate a spatially decoupled mechanical logic: radial localization associated with amplitude‐gradient‐induced drift forces and sustained orbital transport associated with phase‐gradient‐induced angular momentum transfer. This dual‐action mechanism enables a seamless transition from the radial confinement of sub‐wavelength particles (0.02λ) to the fixed‐pivot rigid‐body rotation of wavelength‐scale rods (0.6λ). The observed pivot‐like rotation behavior provides an effective physical route for the orientation and pose control of extended objects, consistent with recent advances in torque‐based structured‐wave manipulation [60].

While the current architecture is based on fixed structural encoding, topological‐charge encoding enables passive chirality control and rotation‐direction reversal without active electronic phase modulation. The frequency‐ and voltage‐dependent responses further reveal the adjustability of orbital dynamics while also indicating a trade‐off between rotational transport speed and orbital‐confinement reliability. Collectively, this passive encoding strategy provides a compact framework for surface‐based structured‐wave manipulation and establishes a basis for future studies of particle handling, object transport, and micromechanical actuation on solid surfaces.

## 4 Materials and Methods

### 4.1 Experimental Setup and Metastructure Fabrication

The experimental platform was designed to generate and characterize flexural‐wave vortex fields on a thin elastic plate. The sample was fabricated from a 2‐mm‐thick aluminum plate (grade 6061) with a total radius of R=90mm. A central manipulation region with a radius of r=26mm of the plate was designated for particle transport. To achieve spatial phase encoding, we integrated 32 labyrinthine phase‐delay units into the plate via precision laser cutting. Each unit consists of a coiled propagation channel that tailors the effective propagation phase delay of the flexural wave through geometry‐induced path modulation. The geometric parameters for a representative unit were $c_{1}$ = 1 mm, $c_{2}$ = 2 mm, $r_{1}=0.25\;\mathrm{mm}$, and l=15mm. By varying the structural height $h_{p}$ from 1 mm to 4.95 mm, a full 2π phase coverage was achieved at the design frequency of 30 kHz. The detailed configuration and the phase‐mapping characterization of the metastructure are provided in Figure S2 (see Supporting Information Appendix).

### 4.2 Excitation and Control System

The vortex field was generated using a ring‐shaped excitation system. A total of 32 rectangular piezoelectric patches (PZT‐5H, 20 mm × 5 mm × 1 mm) were bonded to the top surface and arranged uniformly along the circumference. A computer‐controlled function generator (Rigol DG4162) produced harmonic sinusoidal signals, which were amplified by a high‐voltage power amplifier (Aigtek ATA‐2042) before being applied to the PZT array. The design‐frequency wave‐field characterization was performed at 30 kHz, whereas frequency‐dependent particle‐orbit experiments were conducted at 24–32 kHz under a constant peak voltage of 190 V. Voltage‐dependent particle‐manipulation experiments were performed at peak voltages of 130, 160, and 190 V. The synchronized driving of the transducers, coupled with the structural phase modulation of the labyrinthine units, ensured the formation of a prescribed azimuthal phase distribution $e^{\mathrm{il}\theta }$ with a specific topological charge l.

### 4.3 Wavefield Measurement and Visualization

The out‐of‐plane displacement field W(r,θ) was characterized using a scanning laser Doppler vibrometer (SLDV, LVSC‐400). During the wave‐field measurements, the PZT array was driven by a sinusoidal signal of 10 V peak‐to‐peak (10 $V_{\mathrm{pp}}$). A 36 × 36 mm^2^ area centered on the vortex core was scanned with a spatial step size of 0.2 mm. At each scanning point, the velocity signal was acquired at a sampling rate of 2 MS/s and averaged over three measurements. For harmonic excitation at frequency f, the complex displacement field was obtained from the measured complex velocity field according to W=V/(i2πf). The reconstructed complex wavefield was then used to extract the amplitude envelope |W| and azimuthal phase ϕ. Time‐resolved evolution of the wavefield was visualized to confirm the continuous phase‐driven rotation of the wavefront (see Movie S1).

### 4.4 Particle Manipulation Experiments

Particles (expanded polystyrene spheres with diameters ranging from d = 0.3 to 5 mm) were placed in the central manipulation area. The dynamical response of the particles was recorded using a conventional CMOS camera (Nikon Z30) positioned directly above the plate at 60 fps. Under continuous harmonic excitation, particle confinement near the vortex core and subsequent orbital motion were directly visualized and documented. The rotation velocities and trapping stability were determined by analyzing the recorded video frames. Representative demonstrations of vortex‐field‐induced trapping, OAM‐mediated orbital transport, and multi‐scale manipulation are provided in Movies S3–S5.

## Author Contributions

**Zeyang Bi**: investigation, data curation, validation, formal analysis, writing – original draft, software. **Zhiqiang Li**: investigation, writing – original draft, validation. **Yunkang Ren**: investigation, writing – original draft, validation. **Yugui Peng**: resources. **Xuefeng Zhu**: resources. **Liuxian Zhao**: conceptualization, methodology, investigation, supervision, funding acquisition, writing – review and editing, resources, project administration. **Jun Yang**: resources, supervision, writing – review and editing. **Chuanxing Bi**: resources, supervision, writing – review and editing.

## Conflicts of Interest

The authors declare no conflicts of interest.

## Supporting information




**Supporting File 1**: advs77631‐sup‐0001‐SuppMat.docx.

**Supporting File 2**: advs77631‐sup‐0002‐MovieS1.mp4.

**Supporting File 3**: advs77631‐sup‐0003‐MovieS2.mp4.

**Supporting File 4**: advs77631‐sup‐0004‐MovieS3.mp4.

**Supporting File 5**: advs77631‐sup‐0005‐MovieS4.mp4.

**Supporting File 6**: advs77631‐sup‐0006‐MovieS5.mp4.

**Supporting File 7**: advs77631‐sup‐0007‐MovieS6.mp4.

## Data Availability

The data that support the findings of this study are available on request from the corresponding author. The data are not publicly available due to privacy or ethical restrictions.
