# Supplementary material for: Passive Metasurface Tweezers for Multi‐Scale Orbital Transport and Trapping on Elastic Plates
Source: Adv Sci (Weinh). 2026 Sep 19:e77631. Online ahead of print. doi: 10.1002/advs.77631 (PMC13589307; doi:10.1002/advs.77631)
Supplement: Supplementary file 1 [file ADVS-9999-e77631-s001.docx]

Passive metasurface tweezers for multi-scale orbital transport and trapping on elastic plates

Zeyang Bi^1, 2^, Zhiqiang Li^1, 2^, Yunkang Ren^2,3^, Yugui Peng^4^, Xuefeng Zhu^4^, Liuxian Zhao^2^^,*^, Jun Yang^2,5,*^, Chuanxing Bi^1,*^

^1^Institute of Sound and Vibration Research, Hefei University of Technology, 193 Tunxi Road, Hefei, 230009, China

^2^State Key Laboratory of Acoustics and Marine Information, Institute of Acoustics, Chinese Academy of Sciences, Beijing 100190, China

^3^College of Underwater Acoustic Engineering, Harbin Engineering University, Harbin 150001, China

^4^School of Physics and Innovation Institute, Huazhong University of Science and Technology, Wuhan, China

^5^School of Electronic, Electrical and Communication Engineering, University of Chinese Academy of Sciences, Beijing, 100049, China

**Email:** [zhaoliuxian@mail.ioa.ac.cn](mailto:zhaoliuxian@mail.ioa.ac.cn), [jyang@mail.ioa.ac.cn](mailto:jyang@mail.ioa.ac.cn), [cxbi@hfut.edu.cn](mailto:cxbi@hfut.edu.cn)

Supporting Information Text

**I. Synthesis of Flexural Wave Vortices.** The transverse displacement of a uniform, isotropic thin plate is expressed as $w\left( \mathbf{r},t \right)=Re\{W(\mathbf{r})e^{iwt}\},$ where **r** is the in-plane position vector in cylindrical coordinates, $t$ is time, $W(\mathbf{r})$ is the complex displacement amplitude, and $\omega$ is the angular frequency. According to Kirchhoff–Love plate theory (1). , the frequency-domain displacement field satisfies the governing equation:

$(\nabla^{4}-{k_{b}}^{4})W\left( \mathbf{r} \right)=\frac{F(\mathbf{r})}{D},$	[S1]

where $D=Eh^{3}/\left[ 12\left( 1-\nu^{2} \right) \right]$ represents the flexural rigidity, determined by Young’s modulus *E*, plate thickness $h$, and Poisson’s ratio $\nu$. The parameter $k_{b}$ denotes the bending wavenumber.

Consider *N* piezoelectric transducers acting as point sources, uniformly distributed on a circular ring of radius *R*. The position of the *j*-th source is $\mathbf{r}_{j}=(R,\theta_{j})$, where $\theta_{j}=\frac{2\pi\left( j-1 \right)}{N} (j=1,\ldots,N)$. We assign a phase encoding to each source as $\phi_{j}=l\theta_{j}$. where $l\in Z$ is the prescribed topological charge. The total displacement field at an observation point $\mathbf{r}=(r,\varphi)$ inside the ring ($r<R$) is given by the superposition:

$W\left( r,\varphi\right)=\sum_{j=1}^{N} Q_{0}e^{i\phi_{j}}G\left( \left| \mathbf{r-}\mathbf{r}_{\mathbf{j}} \right| \right),$	[S2]

where $Q₀$ is the source strength and $G\left( \cdot\right)$ is the Green’s function of the plate under point-force excitation.

For sufficiently large $N$, the discrete summation can be approximated by a continuous integral over the source angle (2):

$W(r,\varphi\boldsymbol{)}\approx\frac{NQ_{0}}{2\pi}\int_{0}^{2\pi} e^{il\theta}G\left( \sqrt{r^{2}+R^{2}-2rRcos(\varphi-\theta)} \right)d\theta,$	[S3]

The far-field Green’s function is dominated by its propagating component, which is proportional to the zeroth-order Hankel function of the second kind, ${H_{0}}^{\left( 2 \right)}(\cdot)$, under the $e^{i\omega t}$ time convention. To evaluate the integral analytically, we employ Graf’s addition theorem (3), valid for the interior region $r<R$:

${H_{0}}^{\left( 2 \right)}\left( k_{b}\sqrt{r^{2}+R^{2}-2rRcos(\varphi-\theta)} \right)=\sum_{m=-\infty}^{\infty} {H_{m}}^{\left( 2 \right)}(k_{b}R)J_{m}(k_{b}r)e^{im(\varphi-\theta)},$	[S4]

Substituting Eq. [S4] into Eq. [S3] yields:

$W(r,\varphi\boldsymbol{)}\approx\frac{NQ_{0}}{2\pi}\sum_{m=-\infty}^{\infty} {H_{m}}^{\left( 2 \right)}(k_{b}R)J_{m}(k_{b}r)e^{im\varphi}(\int_{0}^{2\pi} e^{i(l-m)\theta}d\theta),$	[S5]

Using the orthogonality relation $\int_{0}^{2\pi} e^{i(l-m)\theta}d\theta=2\pi\delta_{lm}$ (where $\delta_{lm}$ is the Kronecker delta) the integration acts as a modal filter, ensuring that all harmonics except the one matching the topological charge (*m=l*) undergo destructive interference. Consequently, the synthesized field simplifies to the standard Bessel vortex form:

$W(r,\varphi\boldsymbol{)}\approx A_{l}J_{l}(k_{b}r)e^{il\varphi},$	[S6]

where the complex amplitude coefficient is $A_{l}=NQ_{0}{H_{l}}^{\left( 2 \right)}(k_{b}R)$. Eq. [S6] confirms that the phase of the synthesized field depends solely on the observation angle $\varphi$, establishing a phase singularity at the center (*r=0*) and the well-defined OAM characterized by the integer *l*.

**II. Analytical Derivation of the OAM**

To quantify the dynamical properties of the wavefield, we define the time-averaged in-plane canonical momentum density vector $\mathbf{P}_{\parallel}$as (4, 5):

$\mathbf{P}_{\parallel}=\frac{1}{2}\rho h\omega\mathrm{Im}(W^{*}\nabla W),$	[S7]

where $\rho$ and $h$ are the density and thickness of the plate. By substituting $W\left( r,\varphi\right)\boldsymbol{=}A(r)e^{il\varphi}$ and expanding the gradient operator in cylindrical coordinates ($\nabla={\hat{\mathbf{e}}}_{r}\frac{\partial}{\partial r}+{\hat{\mathbf{e}}}_{\varphi}\frac{1}{r}\frac{\partial}{\partial\varphi}$), we obtain:

$\mathbf{P}_{\parallel}=\frac{1}{2}\rho h\omega\mathrm{Im}\left\{ A(r)e^{-il\varphi}({\hat{\mathbf{e}}}_{r}\frac{\partial A}{\partial r}+{\hat{\mathbf{e}}}_{\varphi}\frac{il}{r}A)e^{il\varphi} \right\},$	[S8]

Since the radial envelope $A\left( r \right)=A_{l}J_{l}(k_{b}r)$ is a real-valued Bessel function, the radial term vanishes upon taking the imaginary part. The momentum density vector is thus purely azimuthal:

$\mathbf{P}_{\varphi}=\frac{1}{2}\rho h\omega\frac{l}{r}\left| A(r\boldsymbol{)} \right|^{2}{\hat{\mathbf{e}}}_{\varphi},$	[S9]

The corresponding OAM density component $L_{z}$ perpendicular to the plate is defined by the *z*-component of $\mathbf{L}=\mathbf{r}\times\mathbf{P}_{\parallel}$:

$L_{z}={(\mathbf{r}\times\mathbf{P}_{\parallel})}_{z}=r\times P_{\varphi}=\frac{1}{2}\rho h\omega{l\left| A(r\boldsymbol{)} \right|}^{2},$	[S10]

Although the instantaneous wavefront propagates according to the phase velocity $v_{p}$ = $-\omega/l$, Eq. (S10) shows that the direction of the azimuthal momentum flux is governed by the sign of the topological charge $l$, whereas its magnitude depends on $\left| l \right|$ and the local field amplitude. This circulating momentum flow provides the physical basis for the observed macroscopic rotation.

**III. Phenomenological Interaction Model for Extended Objects**

For macroscopic objects (e.g., an extended rod), the interaction with the flexural wave is described using a phenomenological interaction framework based on periodic collisions, analogous to the Chladni effect. The object receives time-averaged forces originating from the spatial distribution of the vibration amplitude $\Phi\left( r,\varphi\right)=\left| W\left( r,\varphi\right) \right|$ and the wave momentum.

A. **Radial Trapping: Collision-Induced Drift Force**

Following the stochastic model for Chladni figures (6, 7), the object experiences a drift force proportional to the spatial gradient of the vibration intensity. This radial trapping force $F_{r}$ is:

$F_{r}=-\alpha\Phi\left( r,\varphi\right)\frac{\partial\Phi\left( r,\varphi\right)}{\partial r}=-\frac{1}{2}\alpha\frac{\partial\left| A_{l}J_{l}(k_{b}r) \right|^{2}}{\partial r},$	[S11]

where $\alpha$ is an effective phenomenological coupling coefficient representing the contribution of elastic recovery and frictional dissipation during collisions. The value of α is not directly measured or extracted from the present experiments and is introduced as an effective parameter to describe the radial confinement tendency. Therefore, Eq. (S11) is used to describe the qualitative direction and spatial dependence of the radial confinement force rather than to provide a quantitative prediction of particle trajectories. This restoring force provides radial confinement by driving the object toward a stable equilibrium region defined by the local amplitude gradient of the Bessel field.

B. **Orbital Transport: Azimuthal Vortex Force**

In addition to radial trapping, the object experiences a tangential driving force density magnitude, $\mathbf{F}_{\varphi}$, by absorbing or scattering the azimuthal momentum flux. The direction of this force is intrinsically locked to the azimuthal unit vector ${\hat{\mathbf{e}}}_{\varphi}$. Mathematically, this stems from the helical phase gradient $\nabla\left( l\varphi\right)=(l/r){\hat{\mathbf{e}}}_{\varphi}$. Physically, the helical wavefront provides a time-averaged tangential momentum input during repeated object–plate interactions. Thus, the sign of *l* uniquely determines the chirality of the orbital rotation. Defining $\eta$ as the effective momentum-transfer coefficient (which is not independently calibrated in the present experiments), the azimuthal driving force density is expressed phenomenologically (8):

$\mathbf{F}_{\varphi}=\eta\mathbf{P}_{\varphi}\boldsymbol{=(}\frac{1}{2}\eta\rho h\omega\frac{l}{r}\left| A(r\boldsymbol{)} \right|^{2}){\hat{\mathbf{e}}}_{\varphi},$	[S12]

The *l/r* dependence suggests an intensification of the phase gradient near the center. However, for $\left| l \right|\geq1$ the amplitude vanishes at the origin ($\left| A(r) \right|^{2}\propto r^{2\left| l \right|}$). This competition between geometric enhancement (1/*r*) and amplitude decay ($r^{2\left| l \right|}$) ensures physical continuity: the driving force magnitude $F_{\varphi}$ smoothly vanishes at the singularity, while reaching its optimal balance for driving the object near the trapping ring (intensity maximum).

C. **Total Torque on Extended Objects**

For an extended object occupying an area *S*, the total mechanical torque $\tau_{z}$ about the vortex axis is obtained by integrating the local azimuthal force density over the object surface:

$\tau_{z}=\iint{(\mathbf{r}\times\mathbf{F}_{\varphi})}_{z}dS=\frac{1}{2}\eta\rho h\omega l\iint\left| A\left( r \right) \right|^{2}dS,$	[S13]

Eq. (S13) reveals that the magnitude of the torque is determined by the spatially integrated wave intensity intercepted by the object, while the direction is strictly controlled by the topological charge *l*. This phenomenologically accounts for the experimental observation of reversing orbital rotation by switching the metastructure chirality. For an extended object with finite dimensions, different portions of the object experience different local momentum fluxes due to the radial variation of the vortex field. The resulting spatially distributed momentum transfer generates a net torque around the vortex axis.

The rotational dynamics of the extended object can be qualitatively described by the torque balance:

$\tau_{v\mathrm{ortex}}-\tau_{\mathrm{friction}}=I\frac{d\Omega}{dt},$	[S14]

where $\tau_{\mathrm{vortex}}$ represents the vortex-induced driving torque obtained from Eq. (S13), $\tau_{\mathrm{friction}}$ denotes the dissipative torque arising from the object–plate contact, $I$ is the rotational inertia of the object, and $\Omega$ is the angular velocity. During steady rotation, the angular acceleration approaches zero, resulting in:

$\tau_{v\mathrm{ortex}}=\tau_{\mathrm{friction}},$	[S15]

This balance indicates that interfacial friction, contact area, mass distribution, and surface morphology mainly influence the rotation rate and stability, while the rotation direction remains determined by the sign of the topological charge $l$. Therefore, the proposed model provides a phenomenological explanation for the observed rigid-body rotation of extended objects rather than a fully predictive dynamic model.

**IV. Topological-charge-dependent radial structure and orbital motion**

For an idealized Bessel-type flexural-wave vortex carrying orbital angular momentum, the out-of-plane displacement field can be approximately expressed as:

$W(r,\varphi)=AJ_{l}(k_{b}r)e^{il\varphi},$	[S16]

where $A$ is the field-amplitude coefficient, $J_{l}$ is the Bessel function of the first kind of order $l$, $k_{b}$ is the flexural wavenumber, and $\varphi$ is the azimuthal coordinate. The corresponding radial amplitude distribution is:

$\left| W_{l}(r,\varphi) \right|=A\left| J_{l}(k_{b}r) \right|.$	[S17]

The radial nodal rings are determined by

$J_{\left| l \right|}(k_{b}r)=0,$	[S18]

and the radius of the $n$-th nodal ring is therefore

$r_{l,n}^{node}=\frac{\alpha_{\mid l\mid,n}}{k_{b}},$	[S19]

where $\alpha_{\left| l \right|,n}$ is the $n$-th zero of $J_{\left| l \right|}(x)$. Similarly, the vibration-amplitude extrema satisfy

$J_{\mid l\mid}^{'}(k_{b}r)=0,$	[S20]

and the corresponding peak-ring radius is

$r_{l,n}^{peak}=\frac{\beta_{\mid l\mid,n}}{k_{b}},$	[S21]

where $\beta_{\mid l\mid,n}$ is the $m$-th positive zero of $J_{\mid l\mid}^{'}(x)$.

In the experiments, vortex fields with topological charges $l=+2$ and $l=-2$ were investigated. Because for integer *l*,

$J_{-l}(x)={(-1)}^{l}J_{l}(x),$	[S22]

the two vortex fields have the same ideal radial-amplitude distribution and therefore the same characteristic peak- and nodal-ring radii. However, the phase factors $e^{+i2\varphi}$ and $e^{-i2\varphi}$ have opposite azimuthal winding directions. Reversing the sign of $l$ consequently reverses the chirality of the circulating energy flux and the preferred direction of particle orbital motion, while leaving the radial confinement landscape approximately unchanged.

For $\left| l \right|=2$, the first positive extremum and the first positive zero of $J_{2}(x)$ are $\beta_{2,1}=3.0542$ and $\alpha_{2,1}=5.1356$,

Using the flexural wavenumber of the present structure, the first vibration-peak ring and the first nodal ring are located at

$$r_{l,n}^{peak}=12.41 \mathrm{mm} and r_{l,n}^{node}=20.87 \mathrm{mm},$$

The condition-specific mean orbital radii measured under the investigated particle-size and voltage conditions range from 7.24 to 9.14 mm, as summarized in Table S2. These values are smaller than the theoretical first vibration-peak radius of 12.41 mm, indicating that mean particle orbits are located within the inner annular region between the vortex core and the first amplitude maximum.

**V. Numerical control simulations under different structural-encoding conditions**

To verify that the reconstructed vortex field originates from the designed azimuthal phase encoding, three numerical control simulations were performed, including the no-structure, uniform-encoding, and incorrect-encoding configurations. The same excitation frequency, excitation amplitude, material parameters, and boundary conditions were used in all cases. All control simulations were conducted at the design frequency of 30 kHz.

In the no-structure case, no additional structural phase delay is introduced along the azimuthal direction. Therefore, the phase difference between different azimuthal positions is approximately zero. Similarly, in the uniform-encoding case, all units have the same geometrical parameter and approximately identical transmission phase. As a result, the azimuthal phase gradient is absent, and the generated field cannot satisfy the required azimuthal phase dependence $e^{il\varphi}$. Instead, the wave field mainly exhibits a symmetric field distribution without continuous azimuthal phase winding or a well-defined central phase singularity.

In contrast, the incorrect-encoding case contains structural phase modulation, but the imposed phase sequence does not match the desired azimuthal phase profile. The target azimuthal phase continuity is therefore disrupted, leading to a distorted field with an irregular amplitude distribution and a poorly defined vortex core.

As shown in Supplementary Fig. S4, none of the three control cases reconstructs a regular localized vortex field with a clear central phase singularity, continuous azimuthal phase winding, and annular amplitude distribution. These results demonstrate that the prescribed geometry-encoded azimuthal phase sequence is necessary for reconstructing the target flexural-wave OAM field under single-channel in-phase excitation.

**Fig. S4. Numerical control simulations under different structural-encoding conditions.** The columns correspond to the no-structure, uniform-encoding, and incorrect-encoding cases, respectively. The rows show the phase distribution, instantaneous displacement field $w$, and normalized displacement-amplitude field $\left| W \right|$. The no-structure and uniform-encoding cases mainly exhibit cylindrically symmetric field distributions without continuous azimuthal phase winding, whereas the incorrect-encoding case produces a distorted phase and amplitude distribution with a poorly defined central vortex region.

**VI. Frequency-dependent particle-orbit manipulation and quantitative trajectory analysis**

To support the quantitative discussion in the main text, additional particle-orbit manipulation experiments were performed at different operating frequencies (Movie S3). This section provides the experimental trajectory data and the analysis procedure used to evaluate the manipulation performance under frequency detuning.

Particle-orbit experiments were conducted over the 24–32 kHz range under a constant peak voltage of 190 V, with representative cases at 24, 30, and 32 kHz shown in Fig. S5. Lightweight particles (0.3–0.6 mm in diameter) first underwent a transient aggregation and orbit-formation process following excitation. After the orbital pattern became quasi-steady, a 10-s analysis window from 30 to 40 s was selected for each frequency. For dense-particle dynamics, where long-term identity-preserving tracking of individual particles is hindered by overlaps and collisions, a population-based trajectory analysis was adopted. Particle centroids were detected frame by frame within the region of interest, and short trajectory segments were linked between adjacent frames to estimate local velocities. From these short-track trajectories, the ensemble-averaged orbit radius $R_{\mathrm{mean}}$, radial deviation $R_{\mathrm{std}}$, mean tangential velocity $v_{\theta,\mathrm{mean}}$, and mean angular velocity $\omega_{\mathrm{mean}}$ were extracted.

Fig. S5 shows the representative trajectory-analysis results. The left panels display the detected particle clouds during the selected 30–40 s analysis window, with color denoting time, the red dot marking the vortex center, and the dashed arrow indicating the counterclockwise orbital direction. Bounded counterclockwise motion was observed in the representative recordings at all three frequencies, indicating that vortex-induced particle orbiting can be maintained under the tested detuning conditions. However, the trajectory morphology and rotational speed are clearly frequency dependent; the detuned cases exhibit enlarged orbital radii, altered radial spreading, and reduced angular velocities relative to the 30 kHz design condition.

The right panels of Fig. S5 depict the time-dependent $R_{\mathrm{mean}}$ and $\omega_{\mathrm{mean}}$. $R_{\mathrm{mean}}$ represents the frame-by-frame average distance of all detected particles from the vortex center, while $\omega_{\mathrm{mean}}$ is derived from short-track tangential velocities divided by the instantaneous orbit radius and averaged over all valid samples. Counterclockwise orbital motion is defined as the positive direction, whereas clockwise motion corresponds to negative angular velocity.

**Fig. S5. Frequency-detuned particle-orbit manipulation and quantitative trajectory analysis.** Experimental particle-orbit manipulation results extracted from Supplementary Movie S3 at (A) 24 kHz, (B) 30 kHz, and (C) 32 kHz. For each frequency, the left panel shows the detected particle cloud during the selected analysis window from 30 to 40 s; color denotes time, the red dot marks the vortex center, and the dashed arrow indicates the counterclockwise orbital direction. The inset shows the corresponding excitation frequency and experimental actuator configuration. The right panels show the time-dependent ensemble-averaged orbit radius $R_{\mathrm{mean}}$ and ensemble-averaged angular velocity $\omega_{\mathrm{mean}}$, extracted from the detected particle trajectories. $R_{\mathrm{mean}}$ is calculated as the frame-by-frame average distance of all particles from the vortex center, and $\omega_{\mathrm{mean}}$ is derived from short-track tangential velocities. Counterclockwise orbital motion is defined as positive. Bounded counterclockwise rotation is observed in the representative recordings at all three tested frequencies, whereas both the orbit radius and angular velocity exhibit pronounced frequency dependence.

The quantitative metrics extracted from Fig. S5 are summarized in Table S1. Among the tested frequencies, the 30 kHz design condition exhibits the most favorable overall manipulation response, with the smallest mean orbit radius and the highest angular velocity: $R_{\mathrm{mean}}=10.638$ mm, $R_{\mathrm{std}}=5.254$ mm, $v_{\theta,\mathrm{mean}}=8.504$ mm/s, and $\omega_{\mathrm{mean}}=1.094$ rad/s. Detuning to 24 kHz increases $R_{\mathrm{mean}}$ to 13.951 mm and decreases $\omega_{\mathrm{mean}}$ to 0.586 rad/s. Similarly, at 32 kHz, $R_{\mathrm{mean}}$ increases to 13.001 mm, $R_{\mathrm{std}}$ increases to 7.337 mm, $v_{\theta,\mathrm{mean}}$ decreases to 4.243 mm/s, and $\omega_{\mathrm{mean}}$ decreases to 0.588 rad/s. These results show that bounded orbital motion remains observable under the tested frequency-detuning conditions, while the orbital compactness and rotational speed are most favorable near the 30 kHz design frequency.

Table S1. Quantitative particle-orbit manipulation metrics extracted from Movie S3 under frequency-detuned excitation.

| *f* | Analysis window | Representative time | $R_{\mathrm{mean}}$ (mm) | $R_{\mathrm{std}}$ (mm) | $v_{\theta,mean}$  (mm/s) | $\omega_{\mathrm{mean}}$  rad/s |
| --- | --- | --- | --- | --- | --- | --- |
| 24 kHz | 30–40 s | 37.233 s | 13.951 | 3.534 | 7.868 | 0.586 |
| 30 kHz | 30–40 s | 37.667 s | 10.638 | 5.254 | 8.504 | 1.094 |
| 32 kHz | 30–40 s | 31.167 s | 13.001 | 7.337 | 4.243 | 0.588 |

**Note.** $R_{\mathrm{mean}}$ was calculated as the frame-by-frame average distance of all detected particles from the vortex center. $R_{\mathrm{std}}$ represents the standard deviation of the particle–center distances and was used to characterize the radial spread of the particle distribution. $v_{\theta,\mathrm{mean}}$ was obtained from the short-track tangential velocities, and $\omega_{\mathrm{mean}}$ was calculated from $v_{\theta}/R$and averaged over all valid short-track samples. Positive $\omega_{\mathrm{mean}}$ denotes counterclockwise orbital motion, whereas negative $\omega_{\mathrm{mean}}$ denotes clockwise orbital motion. All reported metrics were calculated over the complete 30–40 s analysis window; any displayed single-frame image was selected only for visualization and was not used independently to determine the reported statistics. The results show that bounded counterclockwise orbital motion is observable at 24, 30, and 32 kHz, while the most favorable manipulation response among the tested conditions occurs near the design frequency of 30 kHz.

**VII. Orbital confinement and voltage-dependent manipulation of particles with different sizes**

To further evaluate the capability of the localized flexural-wave vortex field to manipulate particles over different size ranges, orbital-motion experiments were performed using small particles with diameters of 0.3–0.6 mm and large particles with diameters of 3–5 mm. Representative particle motions under peak excitation voltages of 130, 160, and 190 V are presented in Movie S5. Each experimental sequence was recorded for 40 s. The particle-center positions were extracted using an image-based tracking algorithm, from which the two-dimensional particle trajectories, instantaneous orbital radius $R(t)$, angular velocity $\omega(t)$, and tangential velocity $v_{\theta}(t)$ were calculated. The tangential velocity was determined according to. $v_{\theta}(t)=R(t)\omega(t).$

Fig. S6 presents representative experimental snapshots and the corresponding particle-center trajectories under 130 V excitation. For both particle-size ranges, the extracted trajectories form bounded, nearly closed ring-like paths around the vortex center. These observations indicate that the localized vortex field can drive particles with substantially different sizes into bounded orbital motion. The results therefore demonstrate that the proposed manipulation mechanism is not restricted to a single particle scale.

Because a calibrated external radial force was not applied in the present experiments, an absolute trapping stiffness was not directly determined. Accordingly, the particle behavior is described here in terms of orbital confinement or bounded orbital motion, and the confinement performance is evaluated using the orbital-radius fluctuation, trapping probability, and repeatability across independent experimental trials.

**Fig. S6. Orbital confinement of particles with different sizes in the localized vortex wave field.** (A) Representative snapshots of a small particle with a diameter of 0.3–0.6 mm under 130 V excitation. The red dot denotes the particle center identified by the image-tracking algorithm, and the blue arrows indicate the direction of orbital motion. (B) Particle-center trajectory extracted from a 40 s experimental video for the small particle. The bounded, nearly closed trajectory indicates sustained orbital motion around the vortex center. (C) Representative snapshots of a large particle with a diameter of 3–5 mm under 130 V excitation. (D) Corresponding particle-center trajectory extracted over 40 s. These results demonstrate that the localized vortex wave field can confine particles over different size ranges within bounded orbital regions and drive them into orbital rotation.

For both particle-size groups, increasing the excitation voltage generally increases the angular velocity and tangential velocity. For the small-particle group, the mean angular velocity increases from $1.49\mathrm{rad}\text{ }s^{-1}$at 130 V to $1.91\mathrm{rad}\text{ }s^{-1}$at 160 V and $2.27\mathrm{rad}\text{ }s^{-1}$at 190 V. The corresponding mean tangential velocity increases from $12.09$to $13.70$and $18.45\mathrm{mm}\text{ }s^{-1}$, respectively. For the large-particle group, the mean angular velocity increases from $1.51\mathrm{rad}\text{ }s^{-1}$at 130 V to $1.82\mathrm{rad}\text{ }s^{-1}$at 160 V and $1.99\mathrm{rad}\text{ }s^{-1}$at 190 V, while the mean tangential velocity increases from $10.89$ to $13.91$ and $17.28\mathrm{mm}\text{ }s^{-1}$. These results are consistent with enhanced azimuthal driving as the excitation voltage increases.

The radial response, however, depends on particle size. For the small particles, the normalized radial fluctuations are approximately 0.126, 0.142, and 0.115 at 130, 160, and 190 V, respectively. Thus, the radial fluctuation of the small particles does not exhibit a monotonic voltage dependence. In contrast, the normalized radial fluctuation of the large particles changes from 0.161 at 130 V to 0.154 at 160 V and then increases to 0.201 at 190 V. The large-particle orbit at 190 V is also characterized by a larger mean orbital radius and stronger angular-velocity fluctuation, indicating reduced orbital-confinement stability under the highest tested voltage.

**Fig. S7. Voltage-dependent orbital responses of particles with different sizes.** (A) Time-dependent orbital radius $R(t)$and angular velocity $\omega(t)$of small particles with diameters of 0.3–0.6 mm under peak excitation voltages of 130, 160, and 190 V excitation. (B) Corresponding orbital radius and angular velocity of large particles with diameters of 3–5 mm under the same voltage conditions. The curves were calculated from particle-center trajectories extracted from 40-s experimental videos and processed using a fixed time-window averaging procedure to reduce short-time fluctuations. Increasing the excitation voltage generally increases the angular velocity of both particle groups. The radial response is particle-size dependent: the normalized radial fluctuation of the small particles varies non-monotonically, whereas that of the large particles increases markedly at 190 V, indicating reduced orbital-confinement stability under strong excitation.

**Quantitative evaluation of radial fluctuation, trapping probability, and repeatability**

To provide a quantitative assessment of the orbital-confinement performance and experimental repeatability, each particle-size–voltage condition was independently tested in six trials. For the small-particle group, three single-particle trials and three three-particle trials were conducted, corresponding to 12 tested particles at each voltage. For the large-particle group, three single-particle trials and three two-particle trials were conducted, corresponding to nine tested particles at each voltage.

A particle was classified as successfully confined according to the present operational criterion when it maintained bounded orbital motion around the vortex center for at least 30 s during the 40-s observation period. The particle-level trapping probability was calculated as

$P_{trap}=\frac{N_{stable}}{N_{tested}}\times100\% ,$	[S23]

where $N_{\mathrm{stable}}$ is the number of particles satisfying the sustained-confinement criterion and $N_{\mathrm{tested}}$ is the total number of tested particles.

For each particle-size–voltage condition, the corresponding valid tracking data were combined into a condition-level dataset. The reported orbital radius, angular velocity, and tangential velocity were calculated from this dataset and expressed as the mean value together with the corresponding standard deviation. These standard deviations characterize the overall fluctuations of the particle motion around the mean values during the valid observation period. The normalized radial fluctuation was defined as

$\Delta R=\frac{R_{\mathrm{st}d}}{R_{\mathrm{mean}}} ,$	[S24]

where $R_{\mathrm{mean}}$ and $R_{\mathrm{std}}$ are the mean orbital radius and its corresponding standard deviation, respectively. Experimental repeatability was evaluated separately using the six independent trials and the resulting trapping probability.

The quantitative results are summarized in Table S2. For the small-particle group, the trapping probability decreases from 91.7% at 130 V to 75.0% at 160 V and 66.7% at 190 V. For the large-particle group, the corresponding trapping probabilities are 88.9%, 77.8%, and 66.7%. Therefore, although increasing the voltage enhances the particle angular and tangential velocities, it reduces the probability of maintaining bounded orbital motion for at least 30 s.

The decrease in trapping probability does not necessarily correspond to a monotonic increase in the radial fluctuation for every particle size. In particular, the small-particle group exhibits a non-monotonic variation in $\Delta R$, whereas its trapping probability decreases continuously with voltage. This result suggests that particle loss may also be influenced by intermittent contact dynamics, local defects, particle–particle interactions in the multi-particle trials, and transient excursions that are not fully represented by the time-averaged radial fluctuation alone. For the large-particle group, the degradation of confinement at 190 V is more clearly reflected by the simultaneous increase in the mean orbital radius, normalized radial fluctuation, and angular-velocity fluctuation. These observations indicate that larger particles are more susceptible to orbital destabilization under strong excitation. Within the investigated voltage range, the onset of reduced confinement reliability becomes particularly evident between 160 and 190 V. However, because intermediate voltages were not systematically tested, this range should be regarded as an experimentally observed transition interval rather than a precisely determined escape threshold.

Table S2. Quantitative metrics of particle orbital confinement under different excitation voltages.

| *Particle diameter (mm)* | Voltage (V) | Trials | Tested particles | Successfully confined particlese | $P_{trap}$ (%) | $R_{\mathrm{mean}}$ (mm) | ΔR | $\omega_{\mathrm{mean}}$  (rad/s) | $v_{\theta}$ (mm/s) | *Confinement behavior* |
| --- | --- | --- | --- | --- | --- | --- | --- | --- | --- | --- |
| *0.3–0.6* | 130 | 6 | 12 | 11 | 91.7 | 8.23±1.03 | 0.126 | 1.49±0.30 | 12.09±1.92 | *Bounded orbital confinement* |
| *0.3–0.6* | 160 | 6 | 12 | 9 | 75 | 7.24±1.03 | 0.142 | 1.91±0.29 | 13.70±2.12 | *Reduced confinement probability* |
| *0.3–0.6* | 190 | 6 | 12 | 8 | 66.7 | 8.21±0.94 | 0.115 | 2.27±0.38 | 18.45±2.37 | *Faster rotation with reduced confinement probability* |
| *3–5* | 130 | 6 | 9 | 8 | 88.9 | 7.35±1.18 | 0.161 | 1.51±0.29 | 10.89±1.68 | *Bounded orbital confinement* |
| *3–5* | 160 | 6 | 9 | 7 | 77.8 | 7.89±1.22 | 0.154 | 1.82±0.52 | 13.91±3.42 | *Increased angular-velocity fluctuation* |
| *3–5* | 190 | 6 | 9 | 6 | 66.7 | 9.14±1.84 | 0.201 | 1.99±0.59 | 17.28±3.30 | *Enhanced radial fluctuation and reduced confinement reliability* |

**Note:** Each particle-size–voltage condition was independently tested in six trials. For the small-particle group, three single-particle trials and three three-particle trials were conducted, corresponding to 12 tested particles at each voltage. For the large-particle group, three single-particle trials and three two-particle trials were conducted, corresponding to nine tested particles at each voltage. A particle was classified as successfully confined according to the operational criterion used in this study when it maintained bounded orbital motion around the vortex center for at least 30 s during the 40-s observation period. The trapping probability was calculated as $P_{\mathrm{trap}}=N_{\mathrm{stable}}/N_{\mathrm{tested}}\times100\%$. For each condition, the orbital radius, angular velocity, and tangential velocity were calculated from the corresponding condition-level valid tracking data and are reported as mean ± standard deviation. The standard deviations characterize the overall fluctuations around the mean values during the valid observation period. The normalized radial fluctuation was defined as $\Delta R=R_{\mathrm{std}}/R_{\mathrm{mean}}$. Experimental repeatability was evaluated separately using the six independent trials and the corresponding trapping probability.

**VIII. Qualitative comparison with representative acoustic manipulation strategies**

Table S3 compares the present structurally encoded flexural-wave platform with representative acoustic manipulation strategies. Standing-wave methods are effective for trapping and patterning but provide limited continuous orbital transport. Phased-array systems offer programmable manipulation at the cost of multichannel electronics and synchronization. Guided-wave approaches enable directed surface transport but are constrained by the selected mode and propagation geometry. Acoustic-streaming methods support fluid-mediated particle transport but depend strongly on viscous flow conditions. In comparison, the present platform realizes radial confinement, orbital motion, and passive chirality control under single-channel excitation. Its principal limitation is that the encoded topology is fixed by the fabricated structure and its manipulation performance remains frequency dependent.

Table S3. Qualitative comparison of representative acoustic manipulation strategies.

| Approach | Main mechanism | Excitation/control | Main capability | Principal limitation |
| --- | --- | --- | --- | --- |
| Standing-wave tweezers | Nodes and antinodes | Frequency or geometry | Trapping and patterning | Limited continuous orbital transport |
| Phased-array tweezers | Active phase synthesis | Multichannel electronic control | Programmable manipulation | Electronic and synchronization complexity |
| Guided-wave manipulation | Guided-mode-mediated forces | Mode and waveguide control | Directed surface transport | Restricted by mode and guiding geometry |
| Acoustic-streaming manipulation | Viscous streaming-induced drag | Transducer-driven fluid flow | Collective particle transport | Dependent on fluid and boundary conditions |
| This work | Passive structural encoding of a flexural-wave vortex | Fixed geometry with single-channel excitation; frequency and voltage adjustment | Radial confinement, orbital motion, and chirality control | Fixed encoded topology and dispersion-dependent performance |

**Note.** This comparison is qualitative because the reported systems operate under different physical conditions, including solid-surface and fluid environments, different particle sizes, excitation frequencies, input powers, and performance definitions. Accordingly, the table is intended to clarify the operating principles, control characteristics, representative capabilities, and principal limitations of the different approaches rather than to establish a direct quantitative performance ranking.

SI References

1.	Z. Hou, J. Li, D. Xu, Y. Pei, The multi-frequency vibration metastructure for three-dimensional containerless attractor of particles. *International Journal of Mechanical Sciences* **247**, 108175 (2023).

2.	H. Wang, K. Szekerczes, A. Afanasev, Electromagnetic vortex topologies from sparse circular phased arrays. *J. Phys. Commun.* **6**, 025005 (2022).

3.	C. An, *et al.*, Generation of OAM Beams Using Circular Ring Array With In-Phase Feed. *IEEE Trans. Antennas Propagat.* **71**, 7028–7038 (2023).

4.	K. Y. Bliokh, F. Nori, Spin and orbital angular momenta of acoustic beams. *Phys. Rev. B* **99**, 174310 (2019).

5.	A. T. O’Neil, I. MacVicar, L. Allen, M. J. Padgett, Intrinsic and Extrinsic Nature of the Orbital Angular Momentum of a Light Beam. *Phys. Rev. Lett.* **88**, 053601 (2002).

6.	J. Arango, C. Reyes, Stochastic Models for Chladni Figures. *Proceedings of the Edinburgh Mathematical Society* **59**, 287–300 (2016).

7.	Z. Hou, Z. Zhou, J. Li, Y. Pei, The multi-frequency metastructure vibration motor design for dynamic rotary and morphology manipulation of objects. *Extreme Mechanics Letters* **36**, 100667 (2020).

8.	Z. Hong, J. Zhang, B. W. Drinkwater, Observation of Orbital Angular Momentum Transfer from Bessel-Shaped Acoustic Vortices to Diphasic Liquid-Microparticle Mixtures. *Phys. Rev. Lett.* **114**, 214301 (2015).

**Fig. S1. Schematic of the structurally encoded flexural-wave platform and single-channel excitation configuration.** The circular elastic plate is patterned with an annular arrangement of 32 PZT elements. The PZT array is driven by a single-channel harmonic source through a power amplifier. The electrical traces schematically represent the positive and negative connections of the excitation system. The detailed experimental interconnection of the PZT array is presented in Fig. S3. The required azimuthal phase gradient is generated by the passive serpentine/labyrinthine structural encoding rather than by electronic phase modulation.

Fig. S2. Numerical configuration and performance characterization of the labyrinthine phase-delay units. (A) Geometric configuration of the numerical model for flexural wave vortex manipulation. The circular platform comprises *N*=32 structurally encoded units, arranged to satisfy the azimuthal phase profile for a topological charge of *l*=+2. (B) Magnified view of the meta-units at an azimuthal coordinate $\boldsymbol{\theta}_{\boldsymbol{j}}$, highlighting the structural evolution designed to induce a discretized phase gradient in the counter-clockwise direction. (C) Geometric schematic and design parameters of a single labyrinthine unit cell. (D) Numerically extracted phase delay (blue curve) and transmission coefficient (red curve) of the unit cell as a function of the structural height $\boldsymbol{h}_{\boldsymbol{p}}$. The design achieves a full $\boldsymbol{2}\boldsymbol{\pi}$ phase modulation range while maintaining high-efficiency transmission (>0.75). (E–G) Numerical characterization of the induced flexural wave vortex field (*l*=+2): (E) Perspective snapshot of the transient displacement field $\boldsymbol{W}\left( \boldsymbol{r, \varphi} \right)$ generated by simultaneous excitation from localized PZT sources; (F) Normalized spatial distribution of the displacement amplitude |*W*|, revealing the characteristic central low-amplitude vortex core; and (G) corresponding spatial phase distributio, confirming the conversion of in-phase inputs into a well-defined helical wavefront through passive structural modulation.

**Fig. S3. Experimental configuration and integrated platform for flexural wave vortex manipulation.** (A) Top-view photograph of the meta-plate integrated with an array of ($N=32$) PZT transducers. To achieve a streamlined single-channel control paradigm, the odd-numbered transducers $\left( P1,P3,\ldots,P31 \right)$ are connected in parallel as one interleaved group, whereas the even-numbered transducers $\left( P2,P4,\ldots,P32 \right)$ are connected in parallel as a second interleaved group. Both groups are connected to the same output terminals of a single-channel power amplifier and receive the same harmonic voltage signal, thereby providing spatially distributed, nominally in-phase excitation without independently programmed phase delays. The grouped parallel connection facilitates current and power delivery to the PZT array. The required azimuthal phase gradient for the ($l=+2$) vortex mode is passively encoded through the geometric labyrinthine units. (B) Photographic overview of the experimental setup, comprising a waveform signal generator, a high-voltage power amplifier, and the meta-plate assembly. The platform harnesses harmonic sinusoidal excitation to induce flexural wave propagation in the plate, while the passive structural encoding reconstructs a localized flexural wave vortex field for surface particle confinement and orbital manipulation.

**Fig. S8. Quantitative characterization of chirality-reversed particle motion under negative-topological-charge vortex excitation.** (A) Angular velocity versus time of large particles under different peak excitation voltages (130, 160, and 190 V) with a negative topological charge ($l=-2$). The angular velocity remains negative throughout the observation period, indicating clockwise orbital motion according to the adopted sign convention. (B) Tangential velocity $v_{\theta}(t)$ versus time of large particles under different peak excitation voltages. Increasing the excitation voltage increases the magnitude of the tangential velocity, consistent with enhanced azimuthal driving, while the rotation direction remains unchanged. Together with the positive-topological-charge results presented in the main text, these measurements provide quantitative evidence that reversing the sign of the encoded topological charge reverses the orbital chirality, whereas the excitation voltage primarily regulates the magnitude of the rotational response.

**Fig. S9. Frequency-dependent experimental characterization of the flexural-wave vortex displacement field measured by SLDV at 24, 30, and 32 kHz.** Top row: measured normalized instantaneous out-of-plane displacement fields $w$. Middle row: normalized displacement-amplitude fields $\left| W \right|$, used to identify the central low-amplitude region and extract the corresponding effective vortex-core radius at each frequency. The extracted core radii are denoted by $r_{c,24}$, $r_{c,30}$, and $r_{c,32}$ for 24, 30, and 32 kHz, respectively. Bottom row: phase fields $\phi$, plotted over the range from 0 to $2\pi$, showing the frequency-dependent azimuthal phase distributions and the preservation of the characteristic phase winding associated with the vortex field. The scan area is $36\times36 \mathrm{mm}^{2}$, and the scale bars indicate 10 mm. These direct two-dimensional measurements quantify the frequency-dependent variations in the displacement distribution, phase structure, and effective vortex-core size under detuned excitation.

**Supplementary Movie Legends**

**Movie S1**. **Time-resolved flexural vortex displacement fields under different topological charges and excitation frequencies**.This movie presents the experimentally measured two-dimensional flexural vibration displacement fields obtained by scanning laser Doppler vibrometry (SLDV) on the thin plate. The measured physical quantity is the out-of-plane vibration displacement field under sinusoidal excitation.Part I demonstrates the positive vortex mode with topological charge *l*=+2 at the design frequency of 30 kHz, where the time-resolved displacement evolution reveals the rotating wavefront associated with the encoded helical phase profile.Part II presents the negative vortex mode with *l*=−2. The first sequence shows the vortex displacement field at 30 kHz, demonstrating the reversal of wavefront rotation caused by the opposite azimuthal phase gradient. The subsequent sequences show the corresponding displacement fields at 24 kHz and 32 kHz, respectively, revealing the frequency-dependent evolution of the flexural vortex field under phase under-compensation and over-compensation conditions.

These direct two-dimensional SLDV measurements verify the generated flexural-wave vortex fields, confirm the chirality reversal induced by topological-charge inversion, and demonstrate that frequency detuning modifies the vortex-field distribution while preserving the central vortex structure within the tested operating range.

**Movie S2. Frequency-dependent evolution of flexural vortex-core morphology (24–32 kHz).**

This movie presents the experimentally observed evolution of the flexural vortex-core morphology under frequency detuning from 24 to 32 kHz, utilizing fine sand particles as visual tracers of the low-displacement vortex-core region. Part I ($l=+2$) and Part II ($l=-2$) show the vortex-core morphology at the 30 kHz design frequency, where the structurally encoded phase compensation approaches the prescribed azimuthal phase profile, producing a minimized vortex-core region. Part III presents the low-frequency detuning condition at 24 kHz. The increased flexural wavelength introduces phase under-compensation, leading to an enlarged vortex core and a broader particle accumulation region. Part IV shows the high-frequency detuning condition at 32 kHz. The shortened wavelength results in phase over-compensation, producing an expanded and slightly elliptical vortex-core morphology while preserving the central low-displacement vortex region.

These observations demonstrate that the structurally encoded platform maintains the central vortex structure over a broad frequency range, while the vortex-core size and spatial confinement characteristics remain frequency dependent due to dispersion-induced phase mismatch.

**Movie S3. Frequency-dependent particle orbital motion under broadband flexural vortex excitation.**This movie demonstrates the frequency-dependent orbital motion of particles manipulated by the structurally encoded flexural vortex field over a broad operating frequency range (24–32 kHz). The experiment is performed under a single-channel excitation condition with a peak driving voltage of 190 V and a topological charge of $l=+2$. The manipulated particles have diameters ranging from 0.3 to 0.6 mm.The movie sequentially presents particle orbital motion at different excitation frequencies with a 2 kHz interval (24, 26, 28, 30, and 32 kHz). Each frequency condition records the particle evolution over 0–40 s, revealing the frequency-dependent variation of orbital radius, angular velocity, and confinement behavior.At the 30 kHz design frequency, the structural phase compensation approaches the prescribed azimuthal phase profile, producing the smallest mean orbital radius and the highest measured angular velocity among the tested frequencies. Under frequency detuning toward 24 kHz and 32 kHz, the particles remain in bounded orbital motion, while the enlarged vortex core and modified phase compensation lead to increased orbital radius and reduced rotational velocity.

This movie highlights that the structurally encoded platform preserves vortex-mediated orbital motion across a broad frequency range, while the detailed manipulation performance—including orbital size and rotational dynamics—varies with frequency due to dispersion-induced phase mismatch.

**Movie S4**. **Integrated multi-scale acoustic manipulation and versatile object handling.** This movie demonstrates the multi-scale manipulation capability of the structurally encoded platform for manipulating objects across multiple length scales at 30 kHz (wavelength $\lambda=2.5$ cm, $l=+2$). The sequence begins with the stable orbital rotation of millimeter-scale spheres with diameters of 3–5 mm, where particles with sizes comparable to $\lambda/5$ are confined and driven by flexural-wave orbital angular momentum. This is followed by the dual-mode manipulation of submillimeter particles with diameters of 0.3–0.6 mm, showcasing simultaneous trapping and rotational control at the deep deep-subwavelength scale ($\sim\lambda/50$), which demonstrates subwavelength manipulation enabled by the encoded phase gradient. Finally, a lightweight rolled copper-foil rod with a typical length of approximately 1.5 cm exhibits pivot-like rigid-body rotation around the vortex center, illustrating the extension of the mechanism to wavelength-scale, non-spherical objects. Collectively, these results demonstrate the capability of the platform for multi-scale object manipulation and its potential for integrated microrobotic systems on solid surfaces.

**Movie S5. Orbital motion of small and large particles under different excitation voltages.**
The movie presents representative orbital motions of small particles with diameters of 0.3–0.6 mm and large particles with diameters of 3–5 mm under peak excitation voltages of 130, 160, and 190 V. Each condition was recorded for 40 s. Both particle-size groups exhibit voltage-dependent orbital motion around the vortex center. Increasing the excitation voltage generally enhances the angular and tangential velocities, while higher excitation may reduce the persistence of bounded orbital motion, particularly for larger particles at high voltage. Quantitative analyses of orbital velocity, trapping probability, and repeated-trial statistics are provided in Supplementary Information Section VII.

**Movie S6. Chirality-controlled orbital motion under reversed topological charge excitation.**

This movie demonstrates the passive control of particle rotation direction through structural chirality encoding of the flexural-wave vortex field. The experiments are performed at 30 kHz excitation with a reversed topological charge of $l=-2$, producing clockwise orbital motion of manipulated particles.Part I presents the collective orbital motion of submillimeter particles with diameters of 0.3–0.6 mm. Multiple particles undergo synchronized clockwise rotation around the vortex center, demonstrating the preservation of vortex-induced orbital transport under reversed chirality.Part II shows the orbital motion of a larger single particle with a diameter within the 3–5 mm range. The particle follows a bounded clockwise trajectory, confirming that the chirality-dependent rotational response extends from submillimeter particles to larger objects.

These observations demonstrate that the rotation direction is governed by the sign of the encoded topological charge, while the manipulation mechanism remains effective across different particle sizes without requiring active electronic phase modulation.
